# An Integrated Flexible Bioelectrical and Biochemical Monitoring System Based on Spindle-Structured Directional Sweat-Pumping Nanomesh

**DOI:** 10.1007/s40820-026-02115-w

**Published:** 2026-03-02

**Authors:** Jingzhi Wu, Rongkuan Han, Jianfeng Ma, Jinyi Gong, Tianxin Guan, Peiyan Dong, Hao Tang, Haidong Liu, Jinan Luo, Chang Liu, Yuanfang Li, Degong Zeng, Chuting Liu, Zhikang Deng, Xinyi Qu, Lvjie Chen, Tian-Ling Ren, Jianhua Zhou, Yancong Qiao

**Affiliations:** 1 School of Biomedical Engineering, Shenzhen Campus of Sun Yat-Sen University, No. 66, Gongchang Road, Guangming District, Shenzhen, 518107 Guangdong People’s Republic of China; 2 Key Laboratory of Sensing Technology and Biomedical Instruments of Guangdong Province, School of Biomedical Engineering, Sun Yat-Sen University, Guangzhou, 510275 People’s Republic of China; 3 School of Integrated Circuits, Shenzhen Campus of Sun Yat-Sen University, No. 66, Gongchang Road, Guangming District, Shenzhen, 518107 Guangdong People’s Republic of China; 4 School of Integrated Circuits and Beijing National Research Center for Information Science and Technology (BNRist), Tsinghua University, Beijing, 100084 People’s Republic of China

**Keywords:** Permeable electronics, Nanomesh, Multimodal wearable sensors, Sweat unidirectional transportation, Spindle structure

## Abstract

Wearable epidermal monitoring holds significant importance in health assessment. However, current electronic skins are limited by poor conformability caused by sweat accumulation, discomfort from low breathability and single signal, making long-term, stable, and high-throughput signal recording much challenging. In this study, a spindle-structured directional sweat-pumping nanomesh (SDSN) is developed via electrospinning. By combining multiple asymmetries, including wettability, pore size, and spindle-knots structure, the SDSN establishes synergistic forces that enable unidirectional liquid transport at a rate over 1000 times faster than human sweat production during exercise. To demonstrate the advantages in fluid guidance, a dual-architecture and dual-perspective comparative model framework is constructed. The introduction of Au nanomesh as electrodes allows the Au nanomesh electrode to simultaneously monitor electrochemical and electrophysiological signals, while maintaining excellent skin conformability and motion stability. Additionally, a nanomesh-encapsulated flexible circuit is developed capable of continuous wireless monitoring. This system shows potential for correlation analysis of metabolic energy output and cardiovascular response, making it an ideal tool for health management during intense physical labor and exercise.

**Supplementary Information:**

The online version contains supplementary material available at 10.1007/s40820-026-02115-w.

## Highlights

- Built via controlled electrospinning, the nanomesh integrates wettability and structural gradients, enabling ultrafast unidirectional liquid transport at speeds up to 4.00 mL min^−1^ cm^−2^.
- An Au nanomesh electrode with high breathability and moisture permeability is designed, offering superior conformability and stretchability for stable on-skin monitoring during motion, along with excellent skin compatibility.
- The system enables wireless and continuous electrochemical and electrophysiological monitoring, combining sweat biomarkers and electrocardiogram signals for comprehensive health analysis.

**Supplementary Information:**

The online version contains supplementary material available at 10.1007/s40820-026-02115-w.

## Graphical abstract

**Figure:**
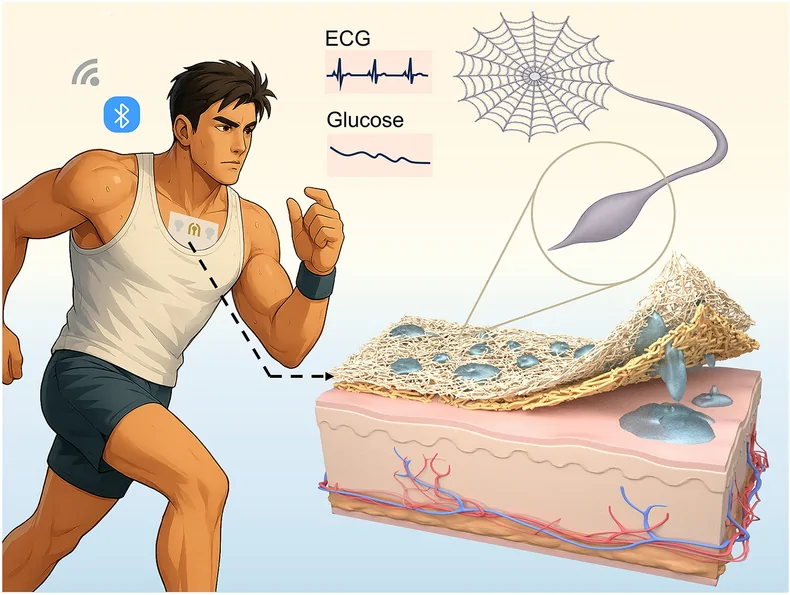

## Introduction

Flexible electronic skin (e-skin) is gradually becoming an important developing trend in the field of sustainable health monitoring due to its stretchability, softness, and high conformity to human skin. However, most conventional e-skins rely on dense substrates such as polydimethylsiloxane (PDMS) and polyethylene terephthalate (PET), which disrupt the thermal-moisture microenvironment at the skin–device interface and degrade thermophysiological comfort during prolonged wear [1]. At rest, the skin releases small amounts of insensible sweat (23 ~ 28 g m^−2^ h^−1^) that should evaporate and diffuse outward. During heat or exercise, the skin discharges sensible sweat (90 ~ 2000 g m^−2^ h^−1^ or 0.15 ~ 3.33 μL min^−1^ cm^−2^), which accumulates as liquid [2, 3]. When the e-skin is impermeable, transport of sweat (as vapor or liquid) from skin to ambient is impeded, leading to heat and humidity buildup, discomfort, and increased risk of irritation and dermatitis [4–8]. In addition, the accumulated sweat film may reduce the electrode adhesion and affect the signal quality [9].

These issues are driving the transition from traditional e-skins to permeable electronics. Porous nanomesh designs now span electrodes [10–14], stress sensors [15–17], stretchable electronics [18, 19], artificial throats [20], sweat sensor [21], etc. However, previous studies primarily rely on the intrinsic porosity of materials to enhance breathability. Nanomesh is typically employed as a passive substrate that maintains vapor-phase sweat transport but inadequately evacuates liquid perspiration, leading to moisture retention at the skin–device interface. Recent studies have leveraged Janus asymmetry to realize spontaneous sweat transport, drawing on bio-inspired surface architectures [22, 23]. Gradients in wettability, pore size, and tapered curvature establish Laplace-pressure differentials that drive liquid toward configurations of lower surface free energy [24–28]. Despite these advances, weak interlayer interactions can undermine durability and system-level integration, such as sensors employing doped conductive networks presents substantial scope for expansion [29–31].

**Fig. 1:**
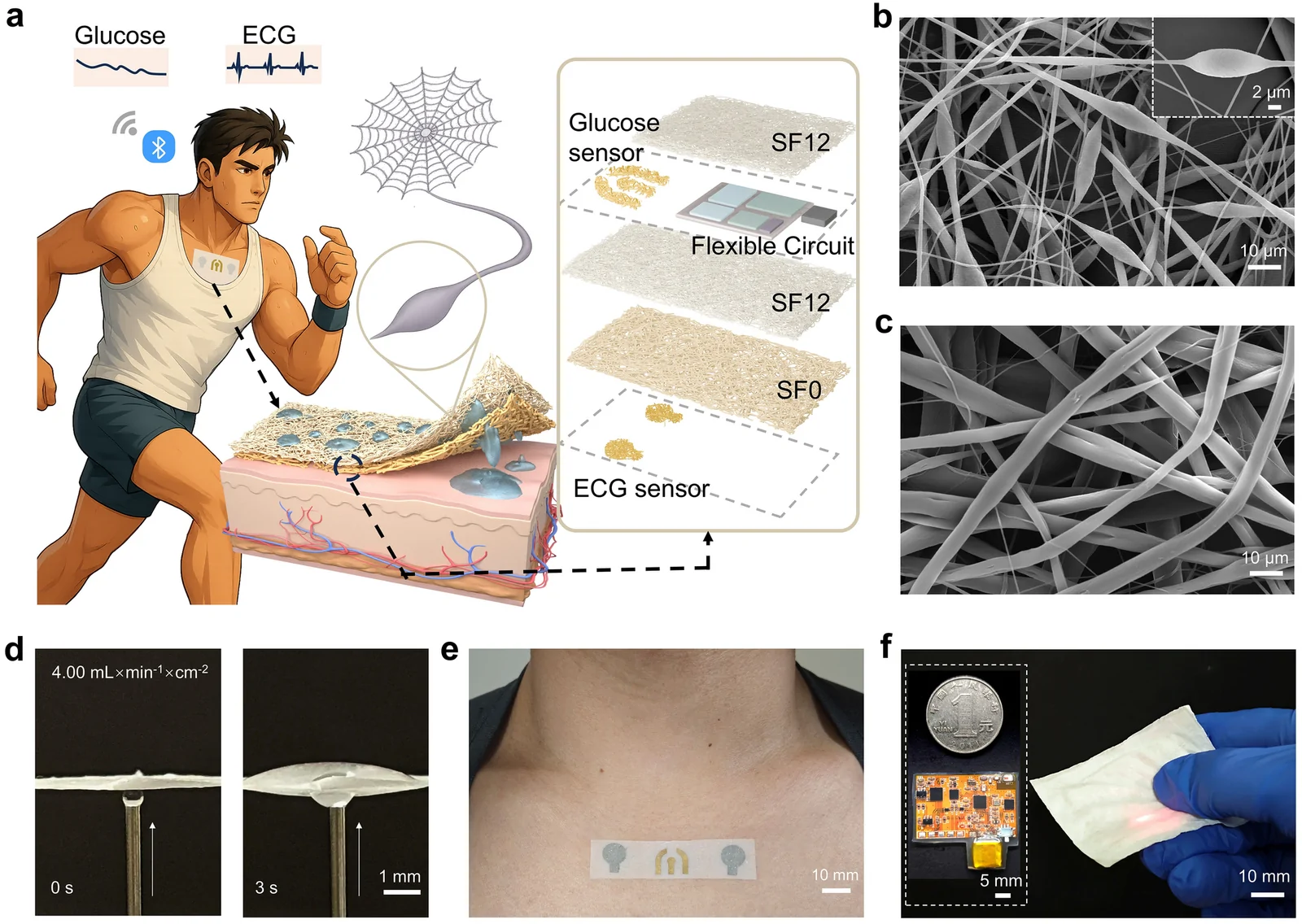
A spindle-structured nanomesh-based flexible system with directional sweat transport for integrated bioelectrical and biochemical monitoring. **a** Schematic diagram of the multimodal monitoring system. **b** Scanning electron microscope (SEM) image of the SF12 layer of the SDSN and the image of the spindle knot. **c** SEM image of the SF0 layer of the SDSN. **d** Anti-gravity water transport through the SDSN under a flow rate of 4.00 mL min^−1^ cm^−2^. **e** Photograph of the ANE on the chest. **f** Photograph of the nanomesh-encapsulated detection circuit

Electrospinning enables continuous, in situ fabrication of nanomeshes, thereby avoiding the weak interlayer interactions common in laminated bilayers. By tuning solute composition, fiber diameter, and membrane thickness, Janus architectures can be programmed [32]. However, engineered fiber-level morphologies have received far less attention. Inspired by spider silk, whose spindle-knot beads generate curvature and surface-energy gradients that direct ambient moisture with reported speeds up to ~ 30 mm s^−1^ [33–36], a spindle-structured directional sweat-pumping nanomesh (SDSN) is fabricated via electrospinning. The hydrophilic layer (SF12, styrene-ethylene-butylene-styrene (SEBS) with 12% Pluronic F127) forms spindle-structured nanomesh, whereas the hydrophobic layer (SF0, SEBS with 0% Pluronic F127) provides a contrasting layer, creating asymmetries in wettability, pore size, and nanomesh morphology. The SDSN delivers a water–vapor transmission rate of 132.6 g m^−2^ h^−1^ and a directional liquid transport capacity of 4.00 mL min^−1^ cm^−2^, approximately 1200 × the typical human sweat secretion rate, thereby supporting unobstructed and long-wear comfort. The impact of spindle-structured layer on water transport performance is independently studied through simulations, with two different models designed: the uniform nanomesh model and the spindle-structured nanomesh model, and results demonstrate a 30% increase in transport speed compared to the model without spindle knots. For groups such as workers, traffic polices, and athletes who are exposed to outdoor environments for a long period of time and have a huge amount of activity, there is an urgent need for new wearable flexible e-skin with excellent breathability and moisture drainage. Given that these populations face higher health risks, relying on a single physiological indicator is often insufficient to evaluate their overall health condition [37, 38]. Instead, multimodal sensors that integrate both physical and chemical signals offer a more comprehensive assessment of body burden and metabolic status, thereby supporting personalized health management [39–41]. Therefore, an integrated system is developed for electrocardiogram (ECG) and sweat glucose monitoring based on the directional sweat-pumping nanomesh (Fig. 1a). In this system, porous Au electrodes are introduced into the SDSN using a sacrificial layer transfer technique. The resulting ultrathin ANE exhibits excellent conformability and stretchability, enabling stable adhesion to the skin during motion and effective suppression of motion artifacts. Equipped with custom-designed electronic circuitry, the system enables wireless transmission of ECG signals and sweat glucose concentrations, offering key physiological insights, including cardiac activity, exercise intensity, and energy expenditure. The integrated multimodal system with directional transport function developed in this study enhances wearer comfort and compliance during high-temperature environments or intense exercise, laying the foundation for future multimodal physiological monitoring applications.

## Experimental Section

### Materials

SEBS is purchased from Kraton. Tetrahydrofuran (THF), glutaraldehyde (GLA), and bovine serum albumin (BSA) are purchased from Macklin. F127 is purchased from Sigma-Aldrich. Polyvinyl alcohol (PVA) is purchased from Aladdin. The glucose oxidase (GOx) is purchased from G-clone. Nafion is purchased from DuPont.

### Preparation of SDSN, ANE, Glucose Sensor

#### Preparations of the SDSN

SF0 refers to an electrospinning solution prepared by dissolving SEBS powder in THF at a concentration of 8 wt%. SF12 represents electrospinning solutions with F127 to SEBS mass ratio of 12%. The solution is continuously stirred for 48 h at 200 r min^−1^ before electrospinning. The double-layer SDSN is obtained by sequential electrospinning of SF0 and SF12, in which the needle model is 18G, the pushing speed is 4 mL h^−1^, the distance between the needle and the receiving plate is 10 cm, the applied voltage is 11 kV for SF0 and 13 kV for SF12, and the electrospinning time for each layer is 40 min.

#### Preparation of ANE

PVA is dissolved in water by heating and stirring at 80 °C to obtain a 10 wt% spinning solution. The PVA electrospun membrane is obtained using a 22G needle to spin for 90 min under the conditions of a pushing speed of 4 mL h^−1^, a receiving distance of 20 cm, and an applied voltage of 15 kV. The Au film is sputtered on the surface of the PVA membrane with a sputtering current of 20 mA for 6 min, giving a thickness of about 100 nm. The pattern of electrodes is performed by laser cutting, and then transferred onto SDSN. Since PVA nanomesh is water-soluble, it can be removed by dissolving in water to finally obtain ANE.

#### Preparation of Glucose Sensor

The GOx is immobilized on the electrode surface through cross-linking with GLA and BSA. A 3 μL aliquot of the prepared mixture, consisting of 1% (v/v) GLA, 1% (w/v) BSA, and 600 U mL^−1^ GOx, is applied onto the working electrode surface and allowed to dry at room temperature for 1 h. Then, 3 μL of 1% Nafion is dropped onto the surface. The reference electrode is fabricated by coating the electrode with silver paste, followed by chlorination via cyclic voltammetry. This process involves scanning two cycles in a solution of 0.1 M HCl and 0.01 M KCl, with a potential range of -0.2~1.0 V at a scan rate of 0.05 V s^−1^.

### Evaluation of SDSN

#### Contact-Angle Measurement

The contact angle is measured using an optical contact angle meter. The sample is fixed on a glass sheet and a drop of 5 μL volume is dropped from above to check the contact angle. The process is recorded with a camera and the contact angle is determined from the image.

#### Anti-Gravity Sweat Transport Characterization

A perspiration block is fabricated by 3D printing (1 cm × 1 cm × 1 cm) with a 5 × 5 array of through-channels (diameter 1 mm). SDSN samples are cut into 1 cm  × 3 cm strips and mounted on a holder, so that the central segment conforms to and covers the channel outlets. Artificial sweat is delivered into the channels using a peristaltic pump at controlled volumetric flow rates. The SDSN continuously transports the incoming fluid from the bottom channels to the top surface. The maximum sustainable volumetric flow rate without backflow is defined as the anti-gravity sweat transfer capacity.

#### Liquid Acquisition and Transport Characterization

*Water harvesting test*: The samples are cut to a size of 1 cm × 3 cm, and the nanomesh is hung on the ground on a 3D printed fixture. The mist from the humidifier is vertically directed at the sample through a conduit. After a period of time, the samples are removed and weighed to measure the amount of moisture collected.

*Capillary uptake test*: The samples are positioned vertically with the lower edge contacting water. The capillary liquid level is recorded as a function of time.

*Lateral diffusion test*: The samples are placed horizontally, a defined droplet is dispensed on the top surface, and the wetted area is tracked over time.

#### Measurement of Nanomesh Parameters

Scanning electron microscopy (SEM) images of the electrospun membranes are analyzed using ImageJ. Thresholding is applied to distinguish fiber regions from pore regions. Nanomesh diameters are measured by drawing line segments, and pore areas are outlined using polygon selections. For each structural feature, 50 samples are marked, and the corresponding parameters are statistically evaluated.

### Evaluation of ANE

#### Gas Permeability Test

The water–vapor transmission rate (WVTR) of the electrode at 37 °C is evaluated. The test is carried out by periodically measuring the weight loss of water in a bottle, where the mouth of the bottle is covered by the sample. WVTR is calculated by the following formula:

1 $${\mathrm{WVTR}} = \frac{\Delta G}{{t \times A}}$$

where *ΔG* is the weight change, *t* is the time during the weight change, and *A* is the area of the bottle mouth.

#### Moisture Permeability Test

The samples are cut into 3 cm × 3 cm squares and placed over a glass slide bearing a 100 μL water droplet. For SDSN samples, the hydrophobic layer is brought into contact with the droplet. The mass loss due to evaporation is recorded at 3-min intervals.

#### Evaluation of Mechanical Properties

Mechanical properties are assessed using a universal testing machine. To evaluate interfacial adhesion, a 90° peeling test is performed on samples (80 μm thickness). The samples are cut into dimensions of 1 cm × 5 cm and adhered to human skin at a peeling rate of 50 mm min^−1^. The same samples are used to measure tensile properties under a stretching rate of 3 mm min^−1^. In addition, the resistance variation of the samples under different tensile strains is evaluated, and cyclic stretching tests are conducted at a fixed strain of 10% for 1000 cycles to assess resistance stability.

#### Skin Reactivity Test

The skin reactivity is evaluated in terms of both skin irritation and thermal conductivity. The gel Ag/AgCl electrode (Silver/silver chloride electrode, SSCE) and ANE are affixed to the arms of three volunteers for a duration of 12 h during their normal daily activities. Following this period, the electrodes are removed, and the covered skin areas are examined for signs of irritation. Additionally, a volunteer wears the electrodes while exercising for 30 min, and the skin temperature post-exercise is monitored using infrared thermography.

#### Skin–Electrode Impedance Measurement

Measurements are performed using the electrochemical workstation. The electrodes are positioned on the arm at a distance of 4 cm, and a voltage of 50 mV is applied, with the frequency range set from 1 Hz to 1 MHz.

#### ECG Acquisition

ECG signals are recorded using physiological data acquisition instruments, following the standard two-lead configuration. ANEs are pre-moistened with pure water and adhered to the subject's skin surface. The ECG after sweating is collected from the volunteer following 30 min of exercise, followed by a 10 min resting period to allow the heart rate to return to a stable level. The SNR is calculated using the following formula:

2 $${\mathrm{SNR}}\mathrm{=}{10}{\mathrm{log}}_{10}\frac{{\mathrm{P}}_{\mathrm{signal}}}{{\mathrm{P}}_{\mathrm{noise}}}$$

where ${\mathrm{P}}_{\mathrm{signal}}$ denotes the mean power during the cardiac activity period of each heartbeat, and ${\mathrm{P}}_{\mathrm{noise}}$ represents the mean power during the quiescent period between heartbeats. The power values are estimated from the data obtained during the ECG test.

#### Glucose Accuracy Comparison Test

A total of 10 sweat samples (1 mL each) are collected from the volunteers and equally divided into two aliquots for experimental and control measurements. The experimental group employs the nanomesh-based glucose sensor integrated with the detection circuit, while the control group utilizes the glucose oxidase method in combination with spectrophotometric analysis as the reference standard. The results from both groups are then compared to assess the accuracy of the proposed sensing platform.

#### Multimodal System Motion Monitoring

The system is attached to the chest area below the volunteer’s clavicle, and the volunteer performs continuous treadmill exercise for one hour. During the activity, the system intermittently collects ECG signals, while sweat glucose monitoring begins 20 min after the onset of exercise to ensure an adequate amount of sweat production. All data are transmitted to a smartphone via Bluetooth in real time. After acquisition, the data are exported and subjected to filtering and denoising processing.

Continuous glucose monitoring (CGM) comparative experiment: The volunteer is equipped with this system and a CGM device (MicroTech Medical, China), performing physical exercise while the system activates electrochemical channels for real-time monitoring. After ten minutes of sweating, the subject consumes a high-sugar beverage and continue exercising. Throughout this period, CGM-derived blood glucose levels and dynamic changes in sweat glucose are recorded.

## Results and Discussion

### Design of the Integrated Multimodal System

The overall concept of the sweat-pumping integrated multimodal monitoring system is schematically illustrated in Fig. 1a, where the SDSN acts as the core functional layer enabling one-way sweat transport. A dense and uniformly distributed spindle-knot structure is created within SDSN. As shown in Figs. 1b, c, and S1, the two layers of the SDSN exhibit distinct morphologies: The hydrophilic SF12 layer possesses smaller pores and periodic spindle knots, whereas the hydrophobic SF0 layer consists of thicker, more uniform nanofibers forming larger pores. When liquid is introduced from the hydrophobic side, it is rapidly pumped upward and retained without backflow (Fig. 1e). Under simulated sweating conditions, the SDSN demonstrates a maximum sustainable anti-gravity liquid flux (Movie S1). Assuming sweat production is stimulated, such as during exercise or by cholinergic agents, the SDSN can withstand flow rates of up to 4.00 mL min^−1^. Leveraging its liquid-diode-like behavior, the SDSN enables simultaneous yet isolated acquisition of electrical and biochemical signals: Sweat is guided to the hydrophilic top layer, minimizing interference during ECG recording, while the upper electrochemical electrodes efficiently capture sweat analytes.

The integrated system is designed for chest-mounted application, which is the clinically preferred site for ECG measurement, ensuring high signal quality while reducing motion artifacts (Fig. 1e). With the incorporation of the ANE, the SDSN-based patch remains flexible and comfortable to wear (Fig. S2). The system further incorporates nanomesh-encapsulated detection circuits and wireless transmission modules (Fig. 1f), maintaining the system’s overall air permeability and water–vapor transmissibility. As demonstrated in Movie S2, stable ECG and sweat glucose signals can be simultaneously recorded during physical activity, highlighting the system’s motion-robust wearability and its advanced capability for real-time multimodal physiological monitoring.

### Design of Spindle-Structured Directional Sweat-Pumping Nanomesh

Electrospinning has emerged as a mainstream route for constructing Janus membranes via compositional integration of dissimilar substrates, thereby enabling directional liquid transport. The technique is attractive for its low cost, simple processing, and scalability to large-area manufacturing. By tuning operating parameters, electrospinning affords precise control over nanomesh architecture (e.g., nanomesh diameter and alignment) without recourse to complex post-processing [42, 43]. Recent developments emphasize coupling multiple asymmetries to achieve superior sweat management and to incorporate additional functions such as sensing (Table S1). Whereas most prior studies have relied chiefly on asymmetries of wettability and pore size, the SDSN architecture introduces an additional dimension, fiber-structural asymmetry, which provides a new direction for enhanced directional liquid transport.

**Fig. 2:**
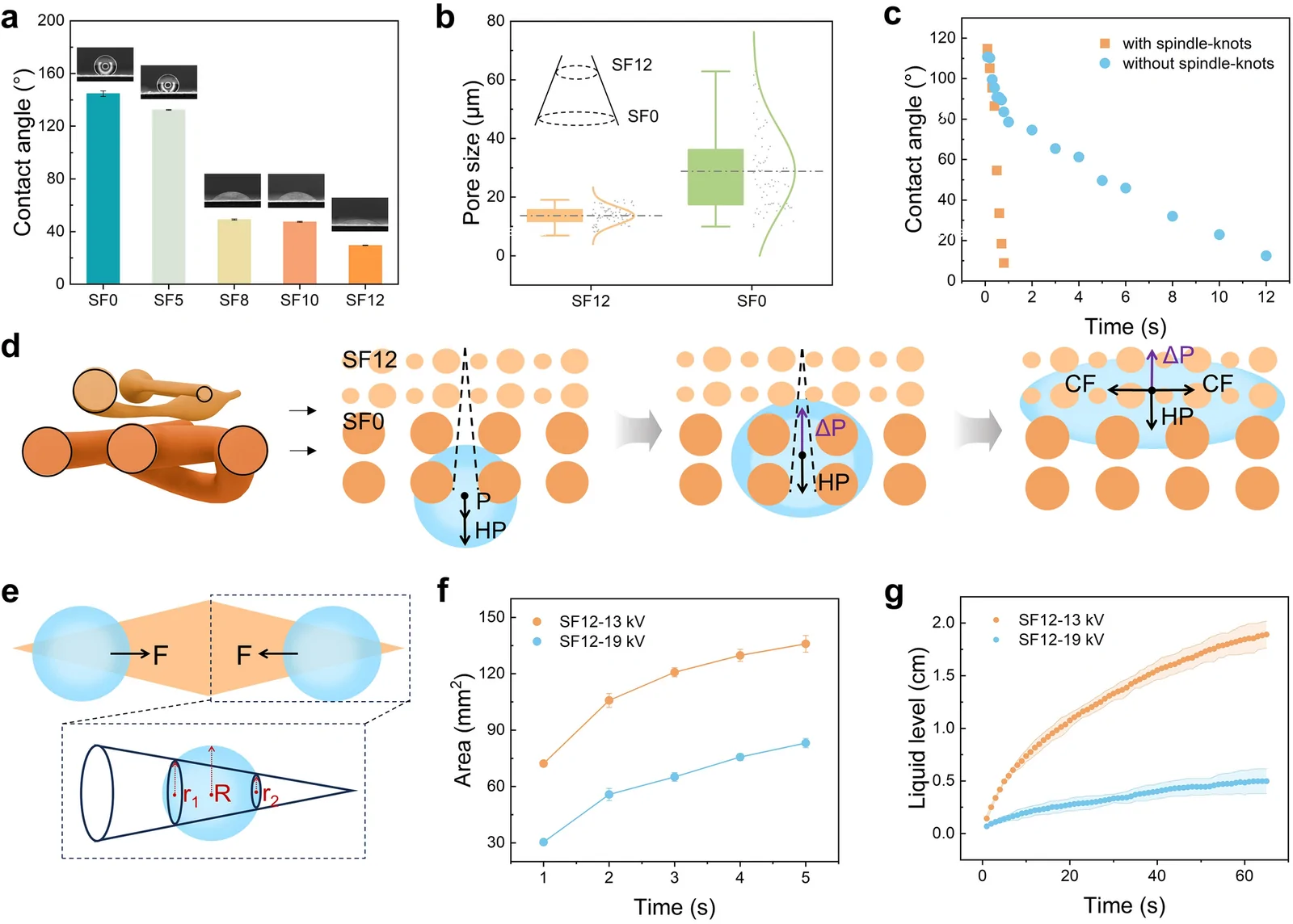
Design of the SDSN and its liquid transport characteristics. **a** Contact angles of nanomeshes with varying SEBS/F127 ratios (SFn denotes SEBS:F127 = (100 − n):n). **b** Pore-size comparison between the SF12 layer and the SF0 layer. **c** Evolution of the contact angle as a droplet penetrates from the hydrophobic layer into the nanomesh. **d** Schematic of the anti-gravity liquid transport mechanism in SDSN. **e** Schematic of directional water transport enabled by structural gradients. **f** Capillary rise (liquid level *vs* time) for hydrophilic nanomesh with and without spindle knots during vertical immersion. **g** Lateral spreading area *vs* time for hydrophilic nanomesh with and without spindle knots

SEBS serves as the SDSN matrix, providing high tensile compliance and good processability. The wettability contrast arises from a polymer blending strategy: Hydrophilic F127 is incorporated into the hydrophobic SEBS solution, which affords more durable modification than post-deposition surface treatments (e.g., spray coating or plasma activation) and avoids performance decay under repeated use. The single-matrix formulation also promotes robust interlayer bonding, reducing the risk of delamination at heterogeneous interfaces. A formulation series (SF0-SF12), in which hydrophilicity increases monotonically with F127 content (Fig. 2a) is examined, and the static water contact angle decreases from 144° (SF0) to 29.5° (SF12). Accordingly, SF12 serves as the hydrophilic layer and SF0 as the hydrophobic layer to establish the desired Janus wettability gradient.

The nanomesh pore size and spindle-knot structure are programmed by systematically tuning electrospinning parameters, including needle inner diameter, applied voltage, and pushing speed. Compared with SF12, SF0 exhibits a larger mean pore size, thereby forming a conical channel (Fig. 2b). The spindle-knot structure is fabricated based on the beading phenomenon commonly observed during electrospinning. As the jet enters the far-field region, electrostatic repulsion between surface charges readily induces instabilities, especially under low applied voltage. Such instability triggers Rayleigh breakup, resulting in the formation of beaded nanomeshes [44, 45]. Although these beads are often considered undesirable in electrospinning, this study transforms such imperfections into valuable features by uncovering the functional advantages of spindle-knot structures in specific applications. At a low applied voltage (13 kV), spindle-knot nanomesh can be reproducibly obtained, whereas increasing the voltage to 19 kV yields spindle-free nanomesh with uniform diameters (Fig. S3). Within this parameter window, pore size and nanomesh diameter distributions remain highly reproducible across batches, as documented in Fig. S4 and Table S2. In addition, film thickness which is governed primarily by deposition time, dictates conformal contact to skin microrelief. Poor skin–device conformality may induce relative motion at the interface and exacerbate motion artifacts [46]. The nanomesh thickness is thus deliberately optimized to suppress such effects. A thickness of ~ 80 μm ensures skin-conforming adhesion, allowing the nanomesh to closely follow skin undulations (Fig. S5).

The SDSN integrates a triple Janus design in wettability, pore size, and nanomesh structure, with the spindle-knot architecture playing a dominant role in transport kinetics. When a 5 μL droplet is applied from the hydrophobic side, the SDSN completes absorption and cross-layer transfer within 0.8 s, yielding a unidirectional transfer rate of ~ 6.25 μL s^−1^ (Fig. 2c). By contrast, the control-group nanomesh that lacks spindle knots requires 13 s for the same volume. The ability of the SDSN to let liquid overcome the hydrophobic layer stems from a surface-energy-driven process: Liquid spontaneously migrates from low-energy (hydrophobic) to high-energy (hydrophilic) regions (Note S1 and Fig. S6). Under perspiration-relevant conditions, although the initial introduction of liquid through the hydrophobic layer encounters resistance from both surface repellency and gravity, the droplets can readily contact the hydrophilic layer. Upon contact, capillary forces dominate, actively drawing the liquid upward against gravity (Fig. 2d). According to the Laplace equation:

3 $${\text{P = (4}}\gamma {\mathrm{cos}}\theta {{)/D}}$$

where *γ* is the liquid surface tension, *θ* is the contact angle, and *D* is the pore size in the nanomesh. Consequently, the SF12 layer generates a strong suction that outweighs the repulsive capillarity of the SF0 layer. Once reaching SF12, the liquid undergoes lateral spreading under capillary action. The spindle-knot structure further amplifies local Laplace pressure via curvature gradients, which promotes in-plane diffusion and lowers the effective resistance for cross-layer pumping, thereby accelerating vertical transport. The principle can be explained by Laplace’s formula, and the difference in forces across the droplet is:

4 $$\Delta F = \gamma \left( {\frac{1}{{r_{2} }} - \frac{1}{{r_{1} }}} \right)$$

where *γ* is the surface tension, while *r*_*1*_ and* r*_*2*_ are the two radii at the spindle junction (Fig. 2e). Since *r*_2_ < *r*_1_, the resultant force vector points from the region of smaller radius to that of larger radius, thereby driving the droplets toward the spindle knot from the slender joint. Experiments corroborate this mechanism: For a 5 μL droplet applied from the hydrophobic side, the SF12 nanomesh with spindle knots (SF12-13 kV) exhibits a significantly higher lateral spreading rate than the spindle-free control nanomesh (SF12-19 kV), which is 60% faster (SF12-13 kV: 135.95 mm^2^ s^−1^, SF12-19 kV: 83.11 mm^2^ s^−1^) (Figs. 2f and S7). Besides, its anti-gravity capillary rise within the nanomesh is also higher, with a 180% faster speed (SF12-13 kV: 0.29 mm s^−1^, SF12-19 kV: 0.076 mm s^−1^), indicating faster vertical pumping kinetics (Figs. 2g and S8). Moreover, the spindle-knot architecture markedly enhances fog collection like spider web, the areal water-harvesting flux increases from 0.082 to 0.174 g cm^−2^ h^−1^ (Fig. S9).

### Dual-Architecture and Dual-Perspective Comparative Model Framework

**Fig. 3:**
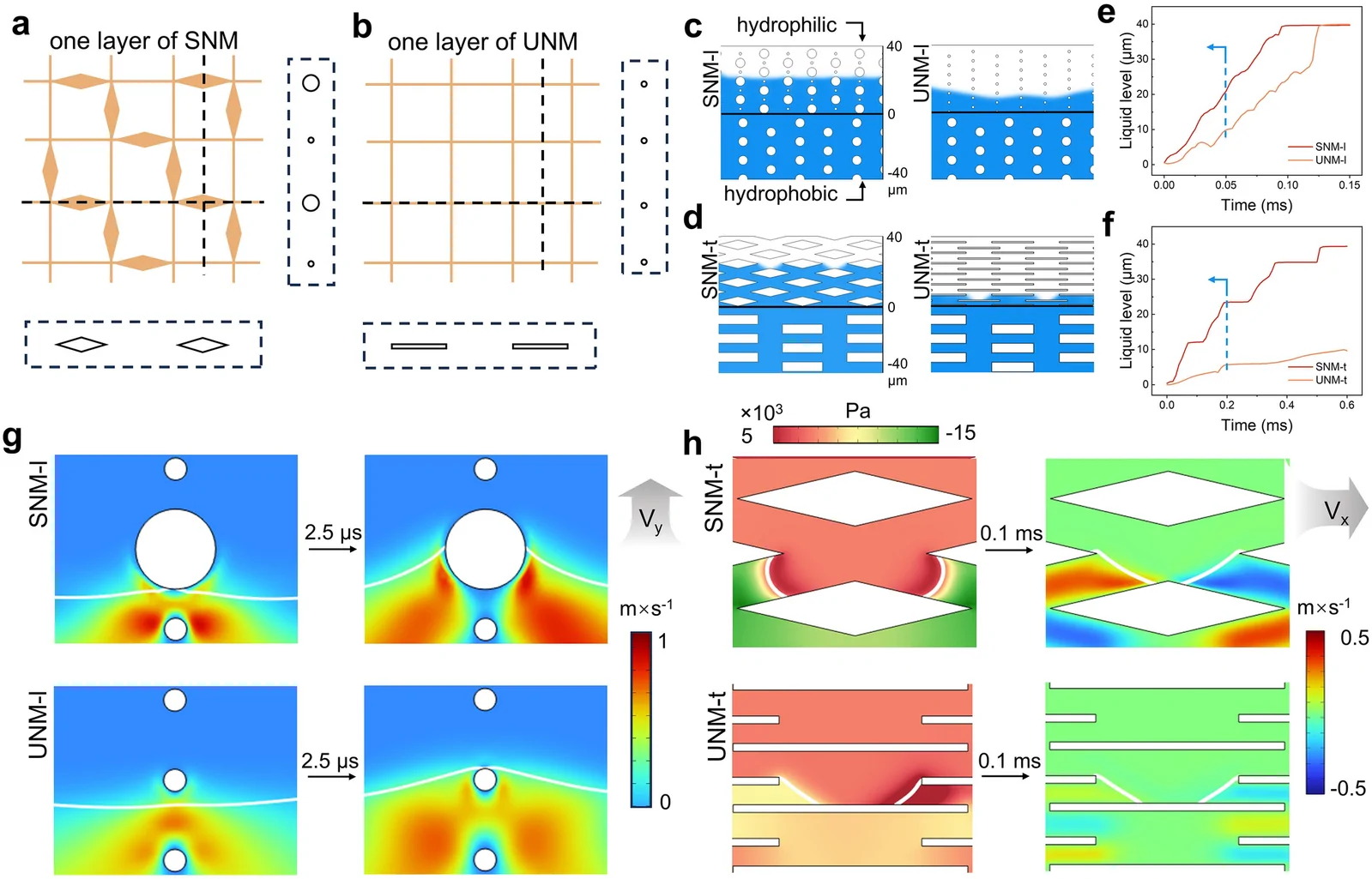
Analysis of dual-architecture and dual-perspective comparative model. **a, b** Structural view of SNM and UNM in the top view, and the schematic unit elements mapped from the two perspectives, respectively. **c** Liquid levels of SNM-l, UNM-l at 0.05 ms corresponding to e. **d** Liquid levels of SNM-t, and UNM-t at 0.2 ms corresponding to f. **e** Comparison of the liquid level over time for SUM-l and UNM-l in liquid anti-gravity transport. **f** Comparison of the liquid level over time for SUM-t and UNM-t. **g** Y-direction velocity distribution at the gas–liquid interface (the white line) in two consecutive frames of SNM-l and UNM-l. **h** Pressure distribution and the x-direction velocity distribution at the gas–liquid interface of SNM-t and UNM-t

As the infiltration and in-mesh dynamics of liquids are difficult to access directly with instrumentation, a dual-architecture and dual-perspective comparative model framework is established to analyze liquid driving at the nanomesh scale. For comparison, two models are constructed: a uniform nanomesh model (UNM) with a spindle-free uniform nanomesh top layer and a spindle-structured nanomesh model (SNM) incorporating a structure designed to enhance water absorption. The electrospun nanomesh membrane is modeled as a multilayered fibrous structure, with nanomeshes in each layer arranged orthogonally, and their diameter and spacing are taken from statistical analysis of SEM images, yielding the top views of both models (SNM: Fig. 3a; UNM: Fig. 3b). We examine both longitudinal and transverse sections (longitudinal perspective: SNM-l and UNM-l; transverse perspective: SNM-t and UNM-t). Upon longitudinal sectioning, the model reveals a cross-sectional architecture composed of regularly arranged circular structures (Fig. 3c, full simulation shown in Movie S3). From the transverse view, nanomeshes are simplified to a rhombic shape for the spindle knot and a rectangular shape for the uniform nanomesh (Fig. 3d, full simulation shown in Movie S4).

Under upward infiltration from the bottom boundary, the SNM outperforms the UNM in both views, yielding a 1.3 times higher velocity in the longitudinal perspective and about 4 times higher in the transverse perspective, consistent with the 1.8-fold enhancement observed in the capillary wicking experiment reported above (Fig. 3e, f). Relative to UNM-l, the rise in liquid level is accompanied by a marked increase in the y-component of velocity along the outer tangential paths of the circular elements in SNM-l, which notably exceeds that in UNM-l (Fig. 3g). This behavior originates from a Laplace-pressure difference established by the liquid bridge formed between the smaller lower circle (joint) and the larger upper circle (spindle knot), biasing flow toward regions of lower curvature and thereby promoting upward transport. In the transverse perspective, SNM-t exhibits a pronounced pressure difference across the gas–liquid interface, indicating that a horizontal Laplace-pressure gradient drives flow toward the spindle knot (Fig. 3h), whereas flow over uniform nanomesh lacks this additional pressure drive. Under this pressure bias, the spindle region in SNM-t exhibits substantially higher transverse velocities than the uniform nanomesh, reaching nearly 5 times instantaneous increase in representative frames, and this micro-scale enhancement contributes to an approximately 1.6-fold macroscopic speed advantage for the spindle-structured nanomesh. Taken together, the simulations and experiments corroborate that spindle knots establish multidirectional capillary surface-energy gradients at the micro-/nanoscale, effectively guiding liquid along prescribed pathways.

### Mechanical and Biocompatibility Properties of the ANE

**Fig. 4:**
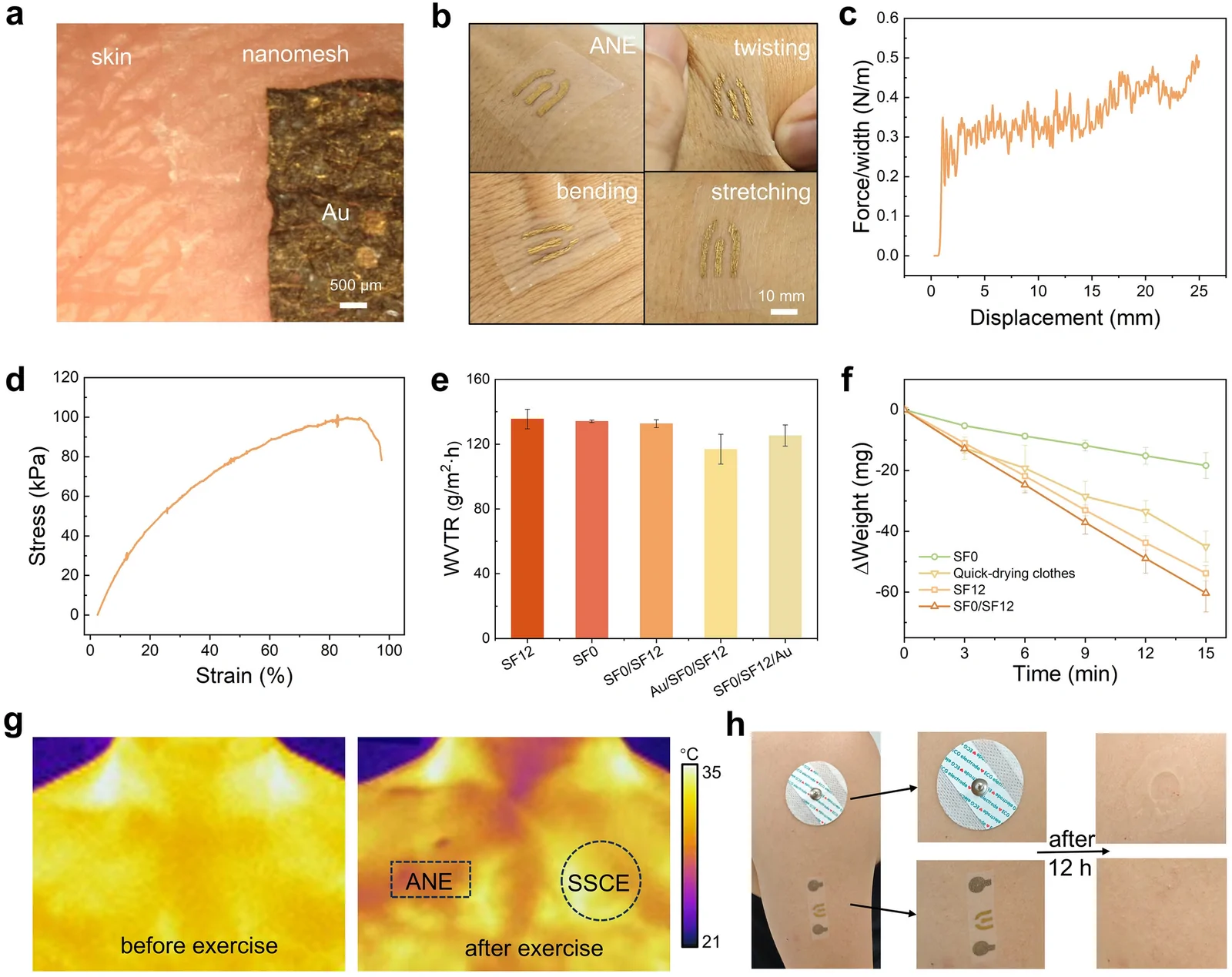
Characterization of the ANE. **a** Photograph of a piece of ANE on the skin. **b** Attachment of the ANE under twisting, bending and stretching conditions. **c** Representative curve of the peeling force of the nanomesh versus displacement. **d** Stress–strain curve of the nanomesh. **e** Comparison of water loss between SF12 (purely hydrophilic nanomesh), SF0 (purely hydrophobic nanomesh), SF0/SF12 (SDSN), Au/SF0/SF12, and SF0/SF12/Au. **f** Comparison of liquid evaporation rates of SF0/SF12 (SDSN), SF12, SF0, and quick-drying clothing. **g** Thermal comfort test of ANE and SSCE. **h** Skin irritation test of ANE and SSCE

Incorporating conductive materials into the SDSN is a critical step toward enabling monitoring applications. Through sputtering, metal deposition can uniformly coat the nanomesh surfaces while preserving the original porous structure and high specific surface area of the nanomesh. Considering that the sputtering process may compromise the surface hydrophilicity of the materials, electrodes are fabricated via a transfer technique. The fabrication of ANE involves a two-step process (Fig. S10). The porous Au electrodes are fabricated by laser patterning Au/PVA film, which is prepared by sputtering onto electrospun PVA membranes. Then, the electrodes are transferred onto SDSN, and the ANE is obtained by removing the PVA sacrificial layer via atomization dissolution. Due to the sweat-pumping capability of the SDSN, moisture transfers from the skin surface to the hydrophilic layer and subsequently flows smoothly along the nanomesh edges under the influence of lateral spread (Fig. S11). The hydrogen bonding and capillary action in sweat ensure that the electrodes adhere closely to the skin (Figs. 4a and S12). In the non-perspiration state, the electrode can adhere to the skin by wetting with water, and after the moisture evaporates, it maintains adhesion through van der Waals forces, which is attributed to its excellent conformability, due to the ultrathin thickness [47] (Movie S5). The ANE maintains stable adhesion to the skin under various deformations, including twisting, bending, and stretching (Fig. 4b). This adhesion can be quantitatively characterized by a peel force of 3 × 10^–3^ N (width = 10 mm, displacement = 10 mm), which exceeds 10 times the elastic force generated at the maximum skin deformation. As a result, the electrode can withstand limb movement and skin strain without spontaneous interfacial separation or displacement, thereby ensuring robustness against motion artifacts (Fig. 4c and Note S2). The electrode in the moist state can remain stable on the skin, the stability of which also comes from the high stretchability of the nanomesh, allowing stretching close to twice its length (Fig. 4d). Under 50% tensile strain, the electrode maintains a resistance variation within 10%, and after 1000 cycles of 10% stretching, the variation remains below 15%, demonstrating its excellent stability under repeated work (Fig. S13).

As measured by the water–vapor permeability test (Fig. 4e), the SDSN exhibits relatively high breathability compared to similar works (Table S3). Moreover, the directional sweat-pumping design does not compromise the permeability of the SDSN as its WVTR is 132.6 g m^−2^ h^−1^, which is similar to the results for the purely hydrophilic or hydrophobic layers (135.5 and 134.0 g m^−2^ h^−1^, respectively). In addition, the WVTR of the nanomesh exhibits a slight decrease but maintains a high level irrespective of the stacking position of the Au electrode layer (Au/SF0/SF12: 116.9 g m^−2^ h^−1^; SF0/SF12/Au: 125.3 g m^−2^ h^−1^). The SDSN demonstrates a significantly enhanced liquid evaporation rate compared to single-layer nanomeshes and quick-drying clothes (Fig. 4f) (SDSN: 267 g m^−2^ h^−1^, SF12: 239 g m^−2^ h^−1^, SF0: 81 g m^−2^ h^−1^), indicating that its sweat-wicking design effectively promotes liquid evaporation, enabling continuous sweat renewal during measurement. High conformability, stretchability, and breathability with moisture permeability are critical for performance under thermohygrometric conditions, endowing the system with potential for monitoring during physical exercise or labor activities.

To enable unrestricted long-term wearability, user-oriented evaluations focusing on thermal comfort and skin irritation are conducted. Benefiting from its efficient sweat transport and evaporation capacity, the ANE exhibits superior thermal comfort during physical activity compared to the SSCE. Infrared imaging reveals that after 30 min of exercise, the skin temperature beneath the SSCE increased by approximately 0.5 °C relative to the surrounding area, whereas the temperature under the ANE decreased by about 1 °C, demonstrating a pronounced evaporative cooling effect (Figs. 4g and S14). The excellent breathability and moisture permeability of the ANE contribute to its outstanding wearing comfort. Subsequently, a 12-h prolonged wear test is conducted with the SSCE and ANE. One of the subjects reported that SSCE caused itching after about 5 h of wear. Upon removal of the electrodes, no signs of irritation or inflammation are observed on the skin covered by the ANE. In contrast, the area under the SSCE exhibited visible erythema (Figs. 4h and S15).

### Application of the System in ECG and Sweat Glucose Monitoring

**Fig. 5:**
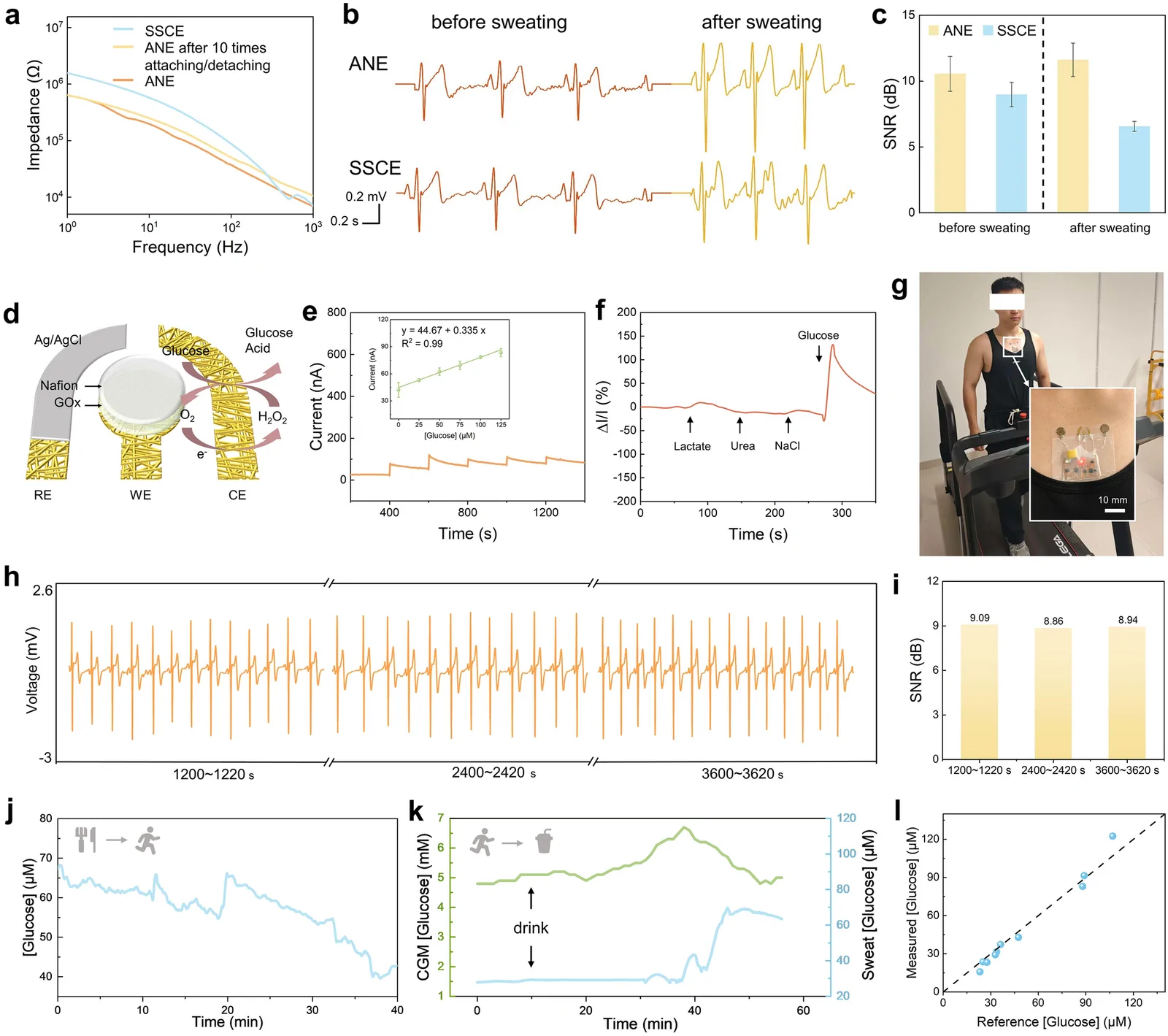
Application of ECG and sweat glucose monitoring. **a** Comparison of the skin–electrode impedance between the SSCE, the ANE and the electrode after 10 times attaching/detaching from skin. **b** ECG recordings collected by the two electrodes before and after sweating. **c** The changes of SNR values of ECG signals in b. **d** Electrochemical three-electrode structure base on ANE. GOx, glucose oxidase. **e** Current response of the sensor within the concentration range of 0~125 μM for glucose and the concentration–current fitting curve. **f** Anti-interference performance of the glucose sensor. The concentrations of lactate, urea, and NaCl are each set to 10 mM, while the glucose concentration is 50 μM. **g** Photograph of the monitoring system on human body. **h** Continuous ECG signals at 20, 40, and 60 min. **i** SNR of ECG signals at 20, 40, and 60 min. **j** Changes in sweat glucose levels during exercise after meal. **k** Changes in sweat glucose levels due to energy supplementation during exercise compared with CGM glucose. **l** Comparison of measured and reference values of sweat glucose

High permeability, conformal skin contact, and low interfacial impedance are decisive for signal integrity in electrophysiology, and the ANE satisfies these criteria with clear performance gains over SSCE. The ANE-skin interfacial impedance is lower than that of SSCE (0.63 vs. 1.57 MΩ, at 1 Hz) and remains low after ten attach-detach cycles (0.64 MΩ at 1 Hz, Fig. 5a), indicating structural robustness and reuse potential. Under perspiration, the ANE preserves waveform fidelity, and the signal amplitude increases due to exercise. *P* waves, QRS complexes, and *T* waves remain clearly resolved. In contrast, the SSCE exhibits baseline wander and a lower signal amplitude (Figs. 5b and S16). Correspondingly, the ANE’s SNR increases from 10.5 to 11.6 dB after exercise, while the SSCE decreases from 9.0 to 6.6 dB (Fig. 5c). These outcomes reflect the breathable, conformal ANE interface that prevents sweat-film formation and decreases the interfacial noise, in contrast to the impermeable gel/adhesive of SSCE. Collectively, ANE delivers superior signal integrity and sensitivity under thermohygrometric stress.

The ANE enables enzyme-mediated sweat-glucose sensing with stable electrochemical behavior (Fig. 5d). Cyclic voltammetry shows a reversible redox pair with a peak-to-peak separation of approximately 135 mV (Fig. S17). The anodic and cathodic peak currents scale linearly with the square root of the scan rate with similar slopes (oxidation: slope = 5.01 × 10^–6^, *R*^2^ = 0.99; reduction: slope =  − 5.49 × 10^–6^, *R*^2^ = 0.99), indicating diffusion control rather than electron-transfer limitation. Amperometric calibration exhibits stepwise current increases with glucose concentration, yielding a sensitivity of 335 nA mM^−1^ (Fig. 5e). The sensor maintains excellent selectivity against common sweat constituents, including interfering species at physiologically relevant sweat concentrations, such as lactate, urea, and NaCl (Fig. 5f). Performance remains stable across typical skin-surface temperatures of 33.5 ~ 36.9 °C and within the sweat pH range of 4.5~6.5, as evidenced by comparable calibration slopes with deviations not exceeding 10% (Figs. S18 and S19). The device is reusable for more than 5 days with consistent responses (Fig. S20).

Bioelectrical and biochemical sensing are integrated with flexible electronics to develop a soft, wireless, and multimodal epidermal system that provides system-level breathability and directional sweat management (Fig. 5g). Specifically, the circuit is encapsulated in a sandwich configuration between the SDSN and the SF12 nanomesh, achieving interfacial self-adhesion through the shared SEBS backbone (Fig. S21). Although the circuit layer is intrinsically impermeable, this adhesive-free textile encapsulation preserves capillary pathways, supporting insensible perspiration through the skin (72.06 *vs.* 28 g m^−2^ h^−1^), while the top hydrophilic layer augments wicking to enable a high liquid-evaporation rate for the encapsulated circuit (143.26 g m^−2^ h^−1^, Table S4). The electronic system is thin and lightweight at 4 mm total thickness and 2.61 g including the battery and Ecoflex waterproof layer. An on-chip Bluetooth module enables continuous real-time wireless transmission from parallel ECG and biochemical front ends (Figs. S22 and S23). The system operates robustly under motion and perspiration, supporting long-duration on-body monitoring.

In the demonstration, the system is affixed to the subject's chest, and both ECG and biochemical sensing data are wirelessly transmitted to a smartphone. Throughout the testing period, the system successfully achieves simultaneous monitoring of ECG signals and sweat glucose concentrations, while demonstrating excellent stability in signal acquisition against motion artifacts and sweat production during physical activity. The ECG data effectively reflected the subject’s physiological state, such as increased heart rate and enhanced signal amplitude in response to cumulative physical exertion (Fig. 5h). The quality of ECG signals remained consistent at 20, 40, and 60 min of physical activity, with the SNR maintained above 90% of the baseline (Fig. 5i). Since the test is conducted after dinner, a decrease in sweat glucose levels following physical activity is observed, indicating postprandial glucose consumption (Fig. 5j). Even when not used for exercise monitoring, the system provides long-duration resting-state ECG monitoring (> 2 h), enabling assessment of cardiac health (Fig. S24). Sweat glucose measurements are able to reflect energy expenditure and metabolic changes in real time. For instance, when the subject consumes a beverage midway through the test, a subsequent rise in glucose level is observed, reflecting dynamic glucose intake and consumption during exercise (Fig. 5k). This trend is consistent with measurements from a commercial CGM device (Fig. S25), with the sweat glucose peak occurring later than the interstitial fluid (ISF) peak. This delay is primarily due to the longer blood-to-sweat lag compared to the blood-to-ISF delay, along with the sweat update time difference [48]. To validate the accuracy of the detection, an additional 10 sweat samples are analyzed and compared using spectrophotometry. Results demonstrated a mean absolute error (MAE) of 4.75 μM and a mean absolute percentage error (MAPE) of 10.61%, indicating that the monitoring system possesses high measurement accuracy (Figs. 5l and S26).

## Conclusions

This study presents a multimodal monitoring system based on the spindle-structured sweat-pumping nanomesh, designed to address the challenges of breathability and sweat management in e-skins, particularly under high-temperature or intensive physical activity conditions. By incorporating spindle knots with curvature gradients into the nanomesh, the system transitions from relying solely on the chemical properties of materials to a synergistic strategy combining microstructural design. It possesses both anti-gravity and in-plane liquid transport capabilities, thereby significantly enhancing directional liquid transport performance and promoting faster sweat release from the nanomesh edges. By embedding structural optimization within the nanomesh itself rather than relying on additional stacked layers, the thickness is effectively constrained to below 100 μm, thereby enhancing skin conformability and providing a foundation for robustness against motion artifacts. The unidirectional transport property of the ANE facilitates active sweat pumping while preventing the reverse infiltration of sweat from other regions into the skin–electrode interface. By mitigating electrode slippage and the associated interference during ECG acquisition, this mechanism enhances signal stability and helps maintain a high SNR. Simultaneously, sweat spontaneously contacts the electrochemical electrode to enable sensing reactions, thereby avoiding direct contact with the skin, which reduces the risk of destruction of the functional layer. Sweat glucose readouts exhibited temporal trends consistent with those of a commercial CGM, while avoiding the invasiveness (indwelling needle ~ 10 mm) and user discomfort inherent to CGM. The SDSN can serve as a platform for developing a comprehensive model to address the sweat refreshment issue, integrating factors such as sweat secretion rate, unidirectional transport, diffusion, and evaporation. With temporal alignment and quantitative calibration, sweat glucose could become a reliable noninvasive surrogate for blood glucose. Consequently, the system provides a comfortable, adaptable wearing experience and enables high-quality, simultaneous acquisition of electrophysiological and biochemical signals, allowing for comprehensive assessment of physiological status and metabolic activity. This technology lays a foundation for the development of next-generation wearable health monitoring platforms that are more integrated, user-adaptive, and intelligent.

## Supplementary Information

Below is the link to the electronic supplementary material.

Supplementary file1 (MP4 6530 KB)

Supplementary file2 (MP4 12596 KB)

Supplementary file3 (MP4 685 KB)

Supplementary file4 (MP4 431 KB)

Supplementary file5 (MP4 4204 KB)

Supplementary file6 (DOCX 15077 KB)
