# Supplementary material for: An Integrated Flexible Bioelectrical and Biochemical Monitoring System Based on Spindle-Structured Directional Sweat-Pumping Nanomesh
Source: Nanomicro Lett. 2026 Mar 2;18:265. doi: 10.1007/s40820-026-02115-w (PMC12953805; doi:10.1007/s40820-026-02115-w)
Supplement: Supplementary file 6 — Supplementary file6 (DOCX 15077 KB) [file 40820_2026_2115_MOESM6_ESM.docx]

Supporting Information for

**An Integrated Flexible Bioelectrical and Biochemical Monitoring System Based on Spindle-Structured Directional Sweat-Pumping Nanomesh**

Jingzhi Wu^1,2^, Rongkuan Han^1,2^, Jianfeng Ma^1,2^, Jinyi Gong^1,2^, Tianxin Guan^3^, Peiyan Dong^1,2^, Hao Tang^1,2^, Haidong Liu^1,2^, Jinan Luo^1,2^, Chang Liu^1,2^, Yuanfang Li^1,2^, Degong Zeng^1,2^, Chuting Liu^1,2^, Zhikang Deng^1,2^, Xinyi Qu^1,2^, Lvjie Chen^1,2^, Tian-Ling Ren^4*^, Jianhua Zhou^1,2*^, and Yancong Qiao^1,2*^

^1^ School of Biomedical Engineering, Shenzhen Campus of Sun Yat-sen University, No. 66, Gongchang Road, Guangming District, Shenzhen, Guangdong 518107, P. R. China

^2^ Key Laboratory of Sensing Technology and Biomedical Instruments of Guangdong Province, School of Biomedical Engineering, Sun Yat-sen University, Guangzhou 510275, P. R. China

^3^ School of Integrated Circuits, Shenzhen Campus of Sun Yat-sen University, No. 66, Gongchang Road, Guangming District, Shenzhen, Guangdong 518107, P. R. China

^4^ School of Integrated Circuits and Beijing National Research Center for Information Science and Technology (BNRist), Tsinghua University, Beijing 100084, P. R. China.

*Corresponding authors. E-mail: [qiaoyc3@mail.sysu.edu.cn](mailto:qiaoyc3@mail.sysu.edu.cn) (Yancong Qiao); [zhoujh33@mail.sysu.edu.cn](mailto:zhoujh33@mail.sysu.edu.cn) (Jianhua Zhou); [RenTL@tsinghua.edu.cn](mailto:RenTL@tsinghua.edu.cn) (Tian-Ling Ren)

**Note S1 Mechanism of droplets orientable transport**

The droplet transport mechanism demonstrates pronounced interfacial dependency. During transport through the hydrophilic layer, droplets are primarily driven by the Laplace pressure gradient (ΔP), which facilitates their capillary infiltration within the nanomesh. The droplets experience vertically oriented hydrostatic pressure (HP). Concurrently, the inherent hydrophilicity induces simultaneous cohesion force (CF) in the lateral dimension, promoting droplets’ horizontal diffusion. Upon approaching the hydrophilic-hydrophobic interface, ΔP undergoes oriented inversion, establishing a transport barrier that equilibrates with HP. The sustained horizontal diffusion maintains a quasi-constant droplet height, thereby minimizing HP and effectively inhibiting droplet penetration into the hydrophobic layer.

In contrast, during hydrophobic layer transport, droplets undergo analogous ΔP-HP interplay. However, the height of droplet accumulates without the horizontal diffusion, resulting in HP escalation. When HP surpasses ΔP, the droplet enabling penetration into the hydrophobic layer. Upon contacting the hydrophilic interface, the synergistic coupling of ΔP and CF facilitating rapid droplet imbibition, thereby establishing a transport from hydrophobic to hydrophilic layer.

**Note S2 The details in ANE characterization**

The classical Kendall's model gives the relation among peel-off force, peeling angle, elastic deformation, and interfacial adhesion energy for an elastic thin-film.

$P=\text{Eh}\left[ \sqrt{{\text{(1-}\text{cosθ}\text{)}}^{\text{2}}\text{+2}\text{∆γ}\text{/}\text{Eh}}\text{-(1-}\text{cosθ}\text{)} \right]$ (S1)

where *P* is the peel-off force per-unit width of the thin-film, $\text{∆γ}$ is the interfacial adhesion energy, $\text{θ}$ is the peeling angle, $\text{E}$ and *h* the Young's modulus and thickness of the film, respectively. Therefore, the 90° peeling force test is used to obtain the minimum peeling force for comparison with the tensile force [S1]. Under a maximum skin strain of 65% [S2], the corresponding elastic force is found to be:

$\text{f}_{\text{e}}\text{=Ehw×0.65=}\text{1.4×}\text{10}^{\text{-}\text{4}}\text{ }\text{N}$ (S2)

Where *E* is Young’s modulus of SDSN (270 kPa), and *h* and *w* are the thickness and the width of nanomesh (*h* = 80 μm, *w* = 10 mm), respectively. The Young's modulus in the peeling force formula is obtained by fitting the linear part of the stress-strain curve, resulting in *y=2.7×x+9.65*, *R^2^=0.99*, from which the Young's modulus *E*=270 kPa can be obtained. The 90° peel test shows that the SDSN exhibits a peel force of 3×10⁻³ N (*w* = 10 mm, displacement = 10 mm), significantly exceeding the elastic restoring force generated at the maximum skin deformation.

**Supplementary Figures and Tables**


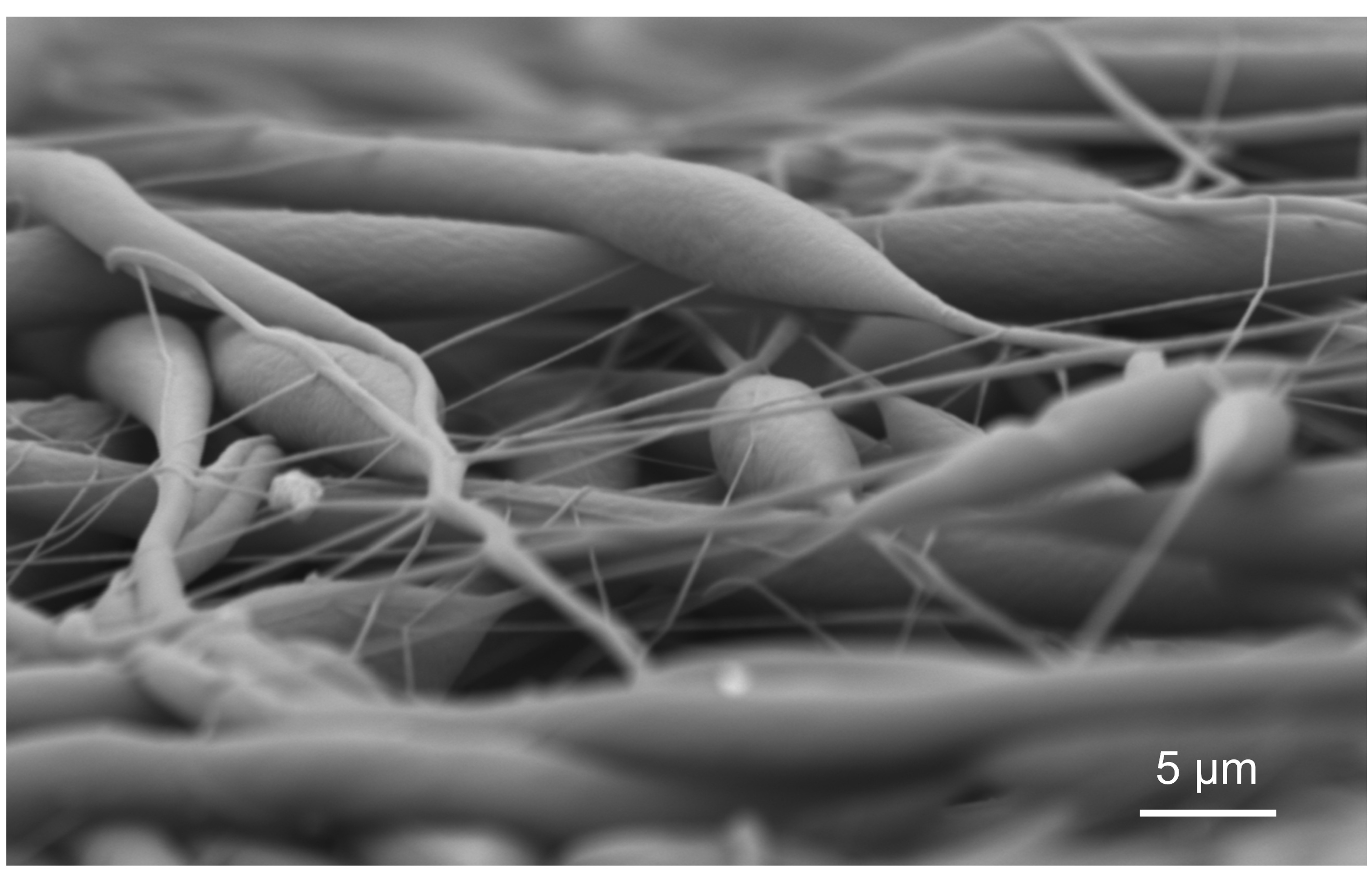


**Fig. S1** SEM of the spindle-knots of SF12 layer taken from the side. The spindle-structured fibers exhibit different orientation patterns, which imparts a porous structure to the nanomesh

**
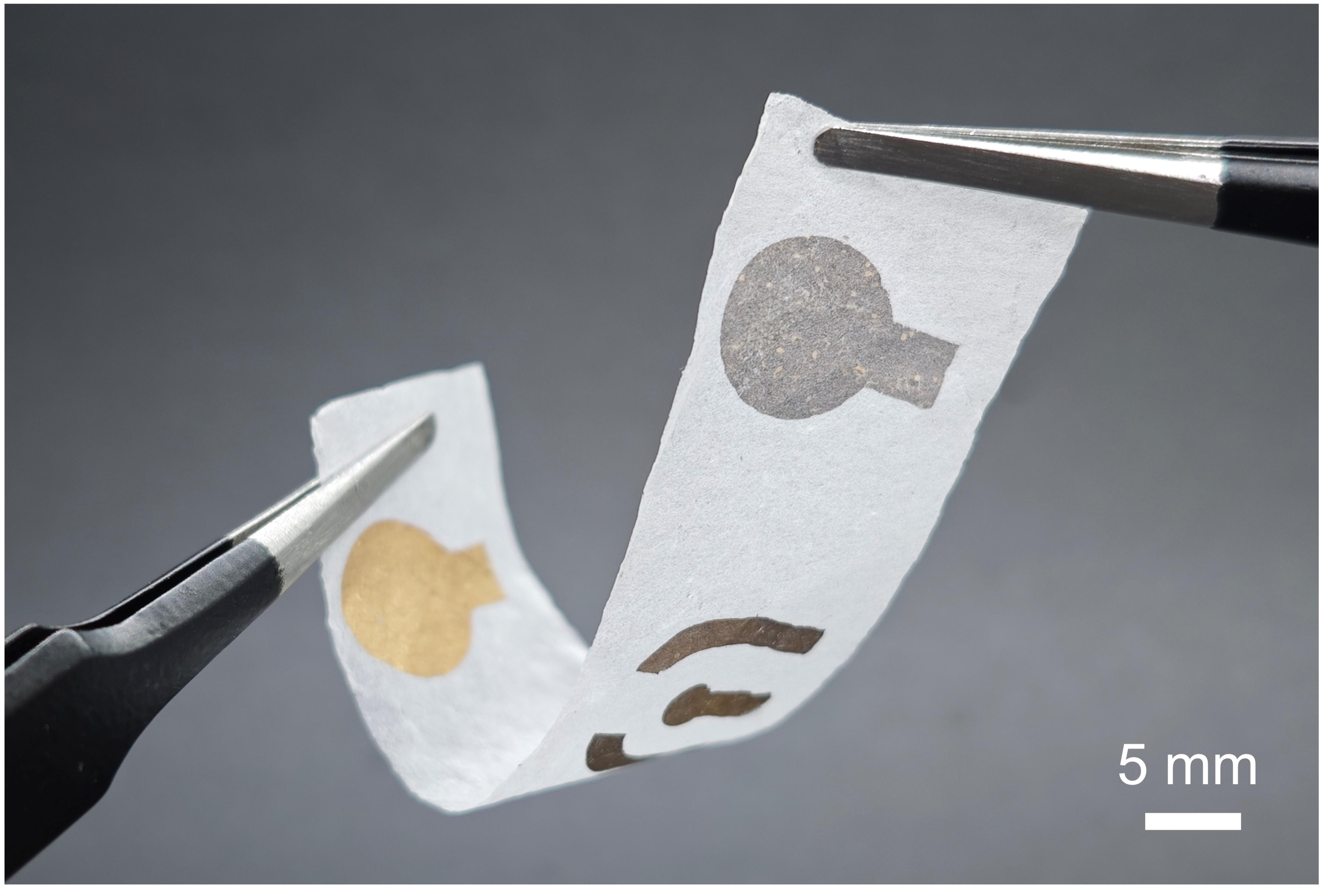
**

**Fig. S2** Photograph of ANE illustrates its flexibility

**
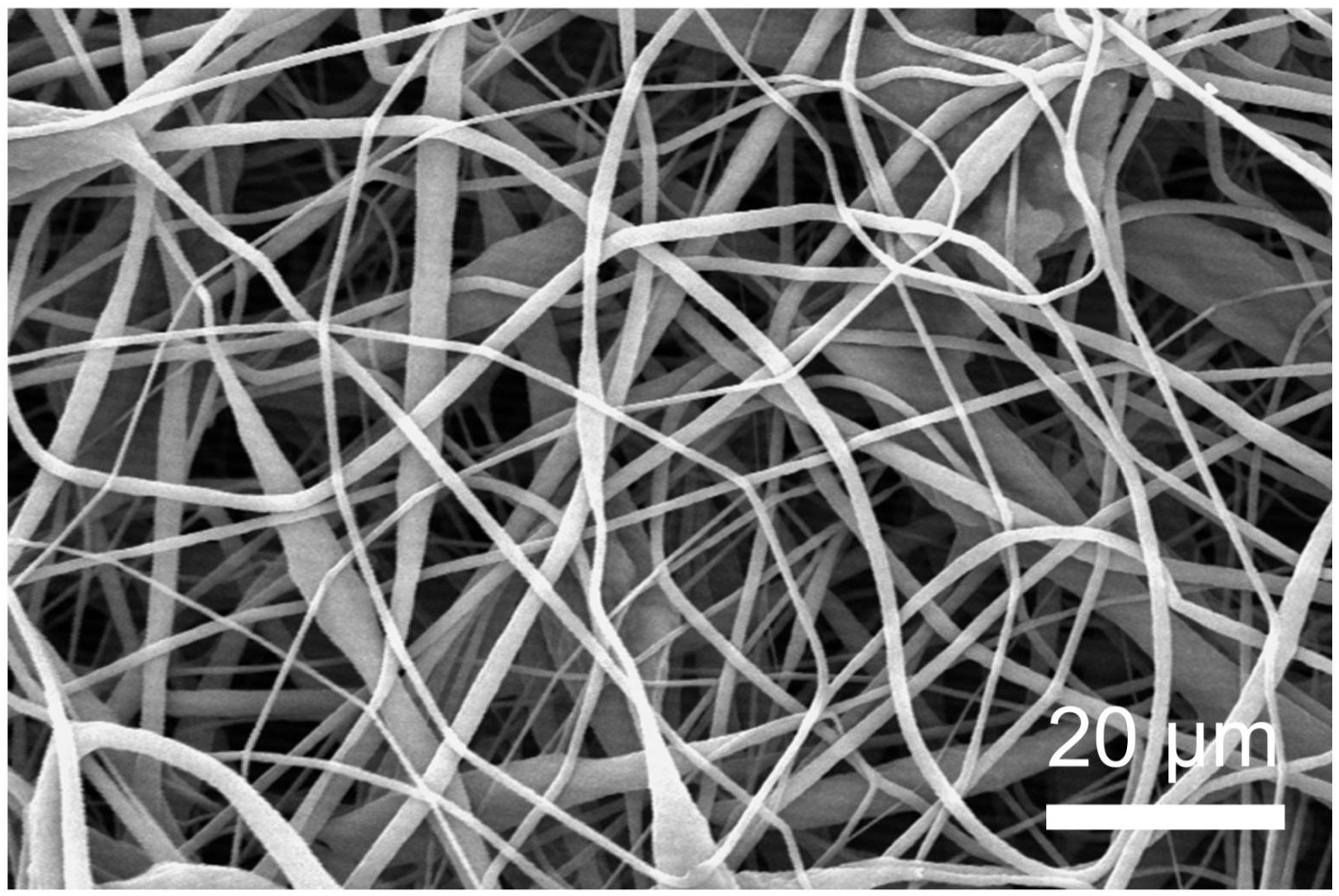
**

**Fig. S3** SEM image of SF12 nanomesh obtained by electrospinning at 19 kV. Under these conditions, with other parameters unchanged, the high voltage induces uniform stretching of the fibers without the formation of spindle-knots structures

**
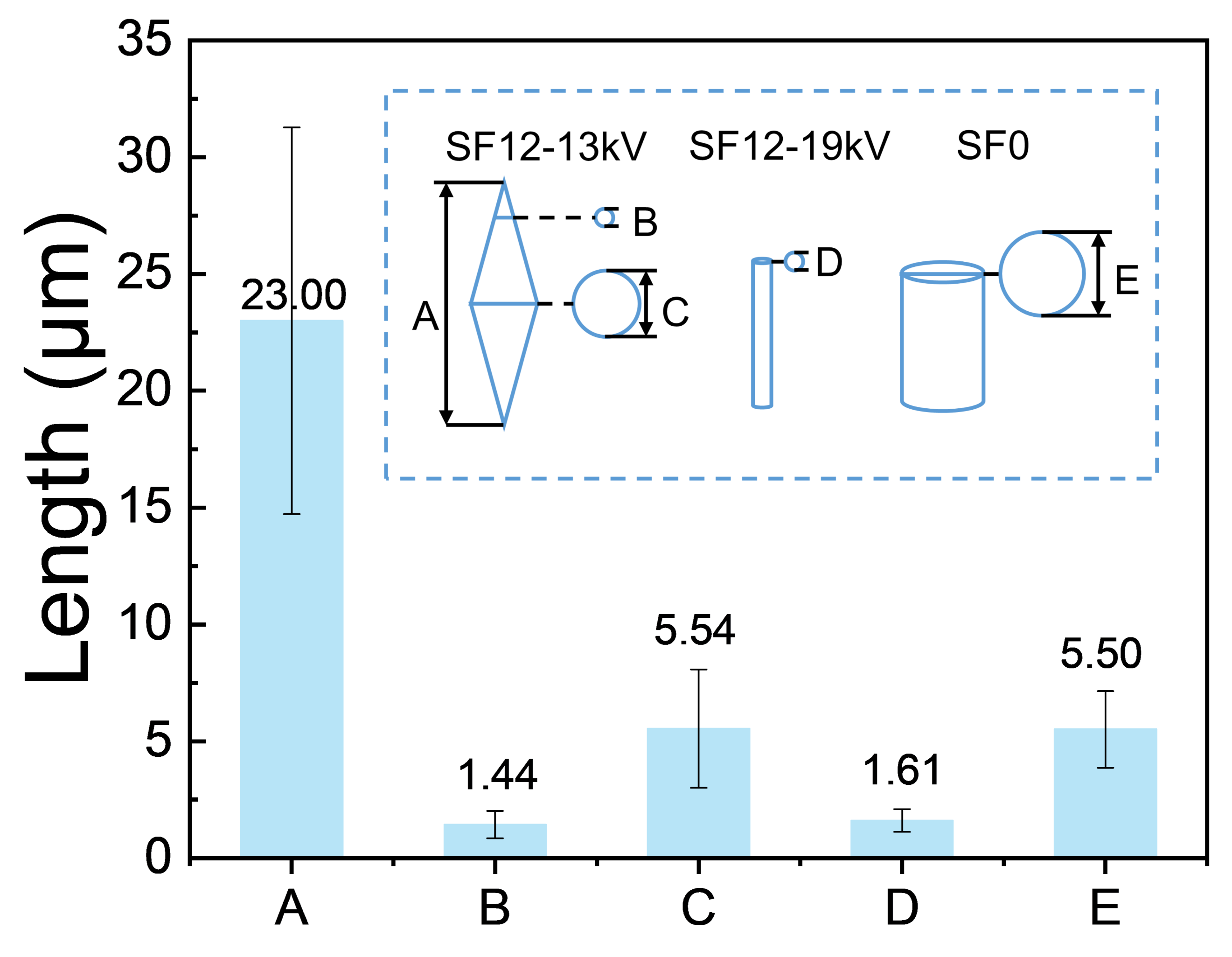
**

**Fig. S4** Measurement of nanomesh parameters from SEM images. A corresponds to the length of a spindle-knot.B corresponds to the diameter at the narrowest point of the spindle-knot. C corresponds to the diameter at the middle of the spindle-knot. D corresponds to the diameter of the spindle-free SF12-19kV layer nanomesh, and E corresponds to the diameter of the SF0 layer fibers


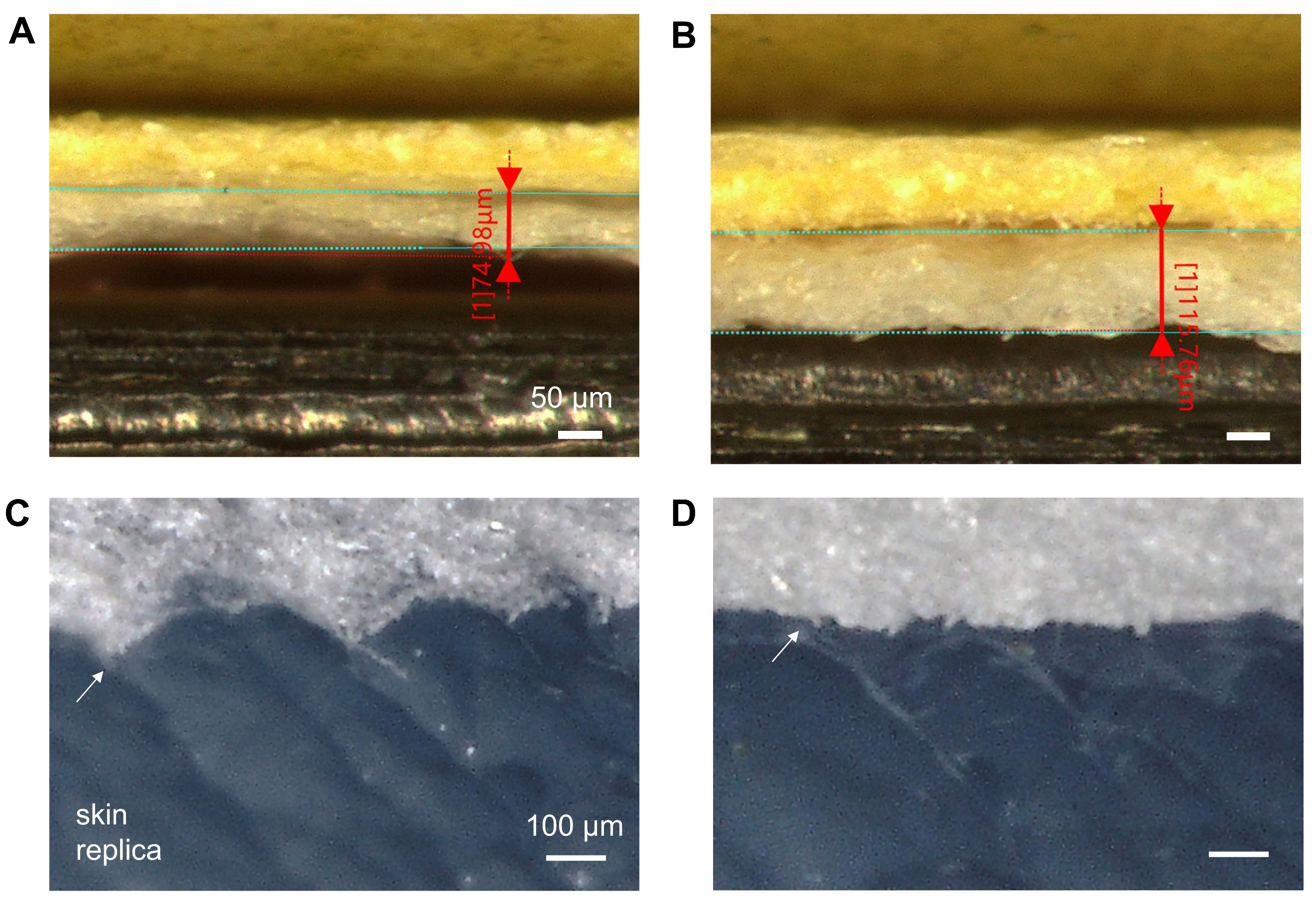


**Fig. S5** Conformability and thickness of SDSN films fabricated with different electrospinning durations. (a) SDSN prepared by electrospinning the SF0 and SF12 layers for 40 min each, yielding a thickness of 74.98 μm. (b) SDSN prepared by electrospinning the SF0 and SF12 layers for 60 min each, yielding a thickness of 115.76 μm. (c, d) Optical micrographs of the thinner film in (a) and the thicker film in (b) attached to a skin replica, respectively. The thinner SDSN conforms more closely to the skin microtopography and bends more readily, indicating superior conformability


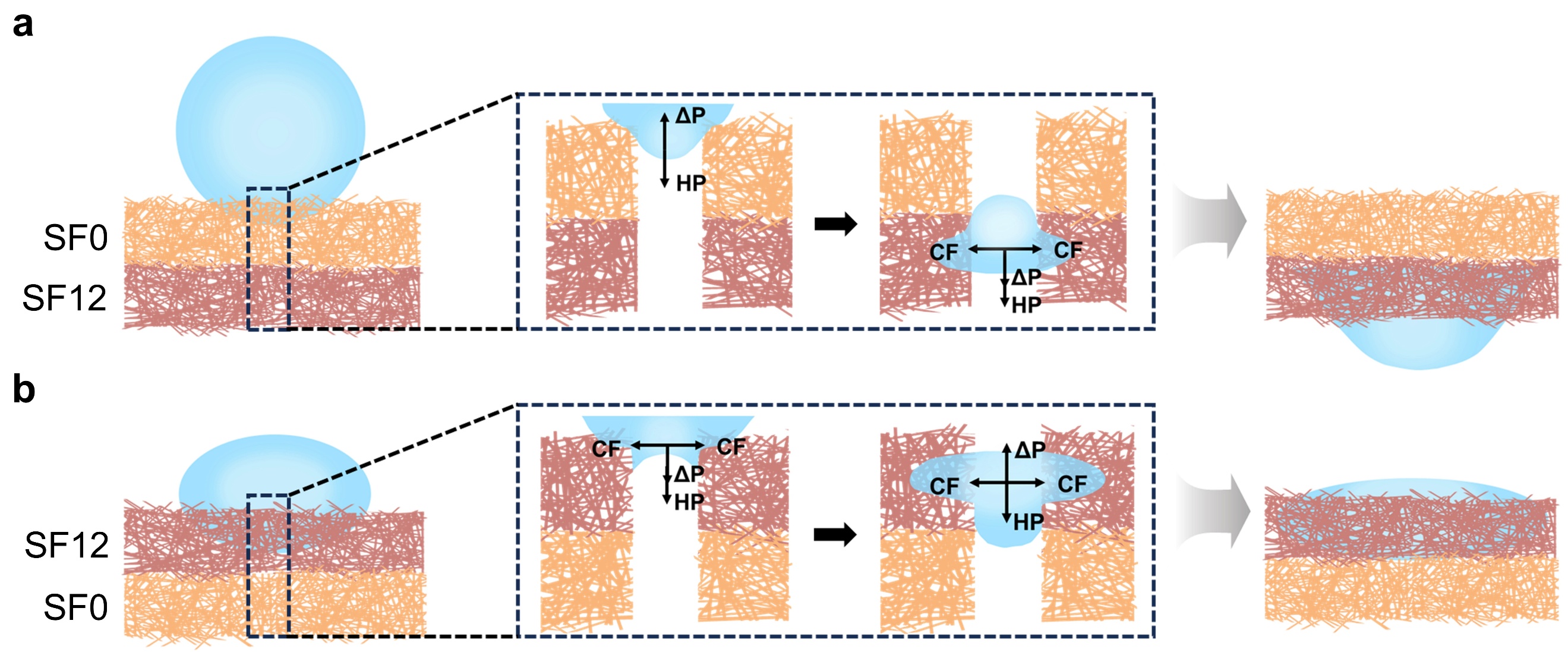


**Fig. S6** Schematic of the principle of unidirectional transport in SDSN. (a) Liquid enters the hydrophilic layer from the hydrophobic layer. (b) Liquid enters the hydrophobic layer from the hydrophilic layer


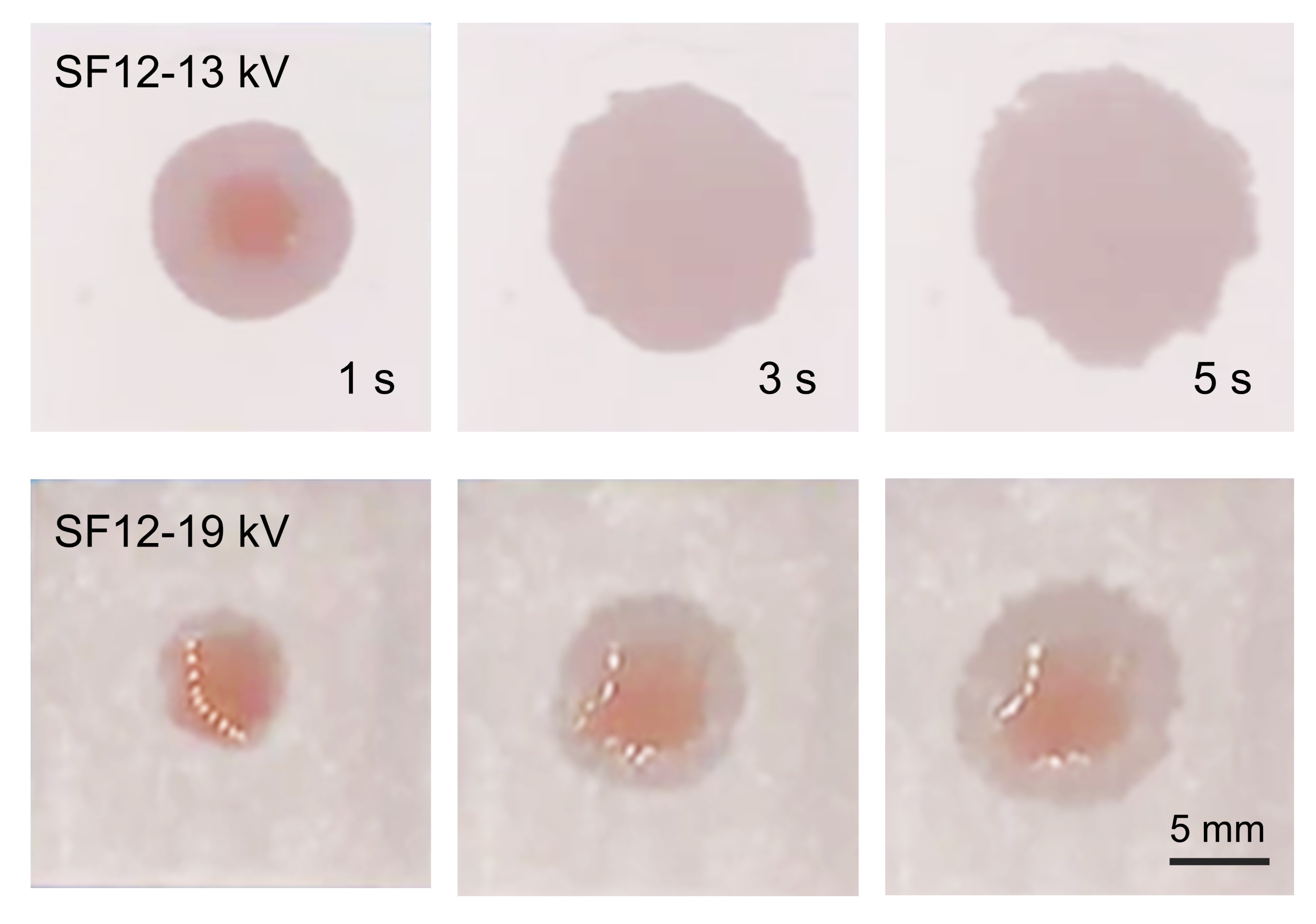


**Fig. S7** Comparison of the lateral diffusion speed of liquid on hydrophilic nanomeshes with and without spindle knots


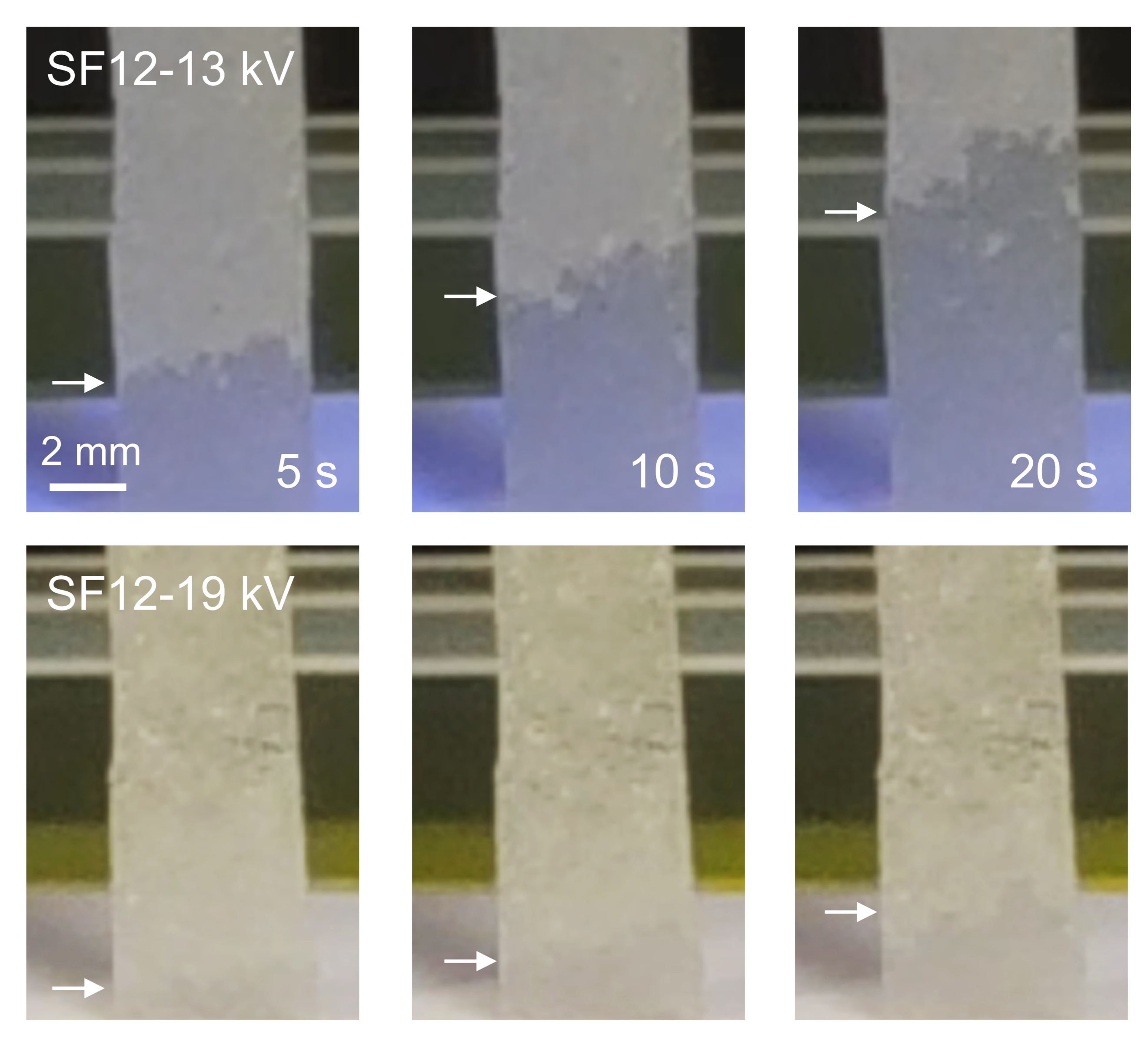


**Fig. S8** Comparison of anti-gravity capillary wicking velocity on hydrophilic nanomeshes with and without spindle knots

**Fig. S9** Comparison of water collection performance with and without spindle-knots

**
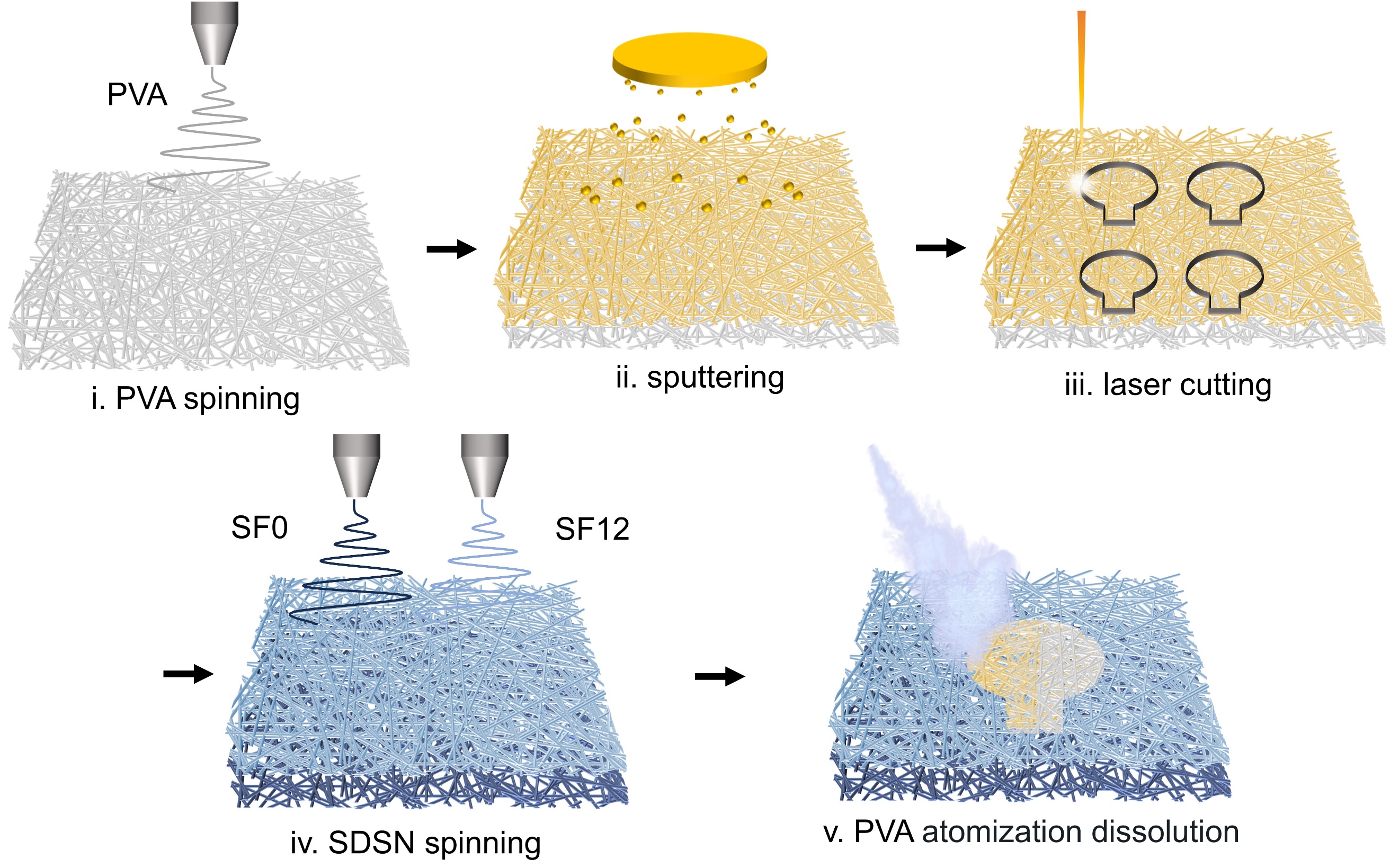
**

**Fig. S10** Preparation process of ANE. Au electrodes are developed on the nanomesh using a PVA sacrificial layer transfer method: an Au thin film is sputtered onto the surface of the PVA spun membrane, patterned using laser cutting. Subsequently, the SDSN is prepared via electrospinning, and then Au/PVA is transferred onto the SDSN. Finally, the PVA layer is removed by atomization dissolution

**
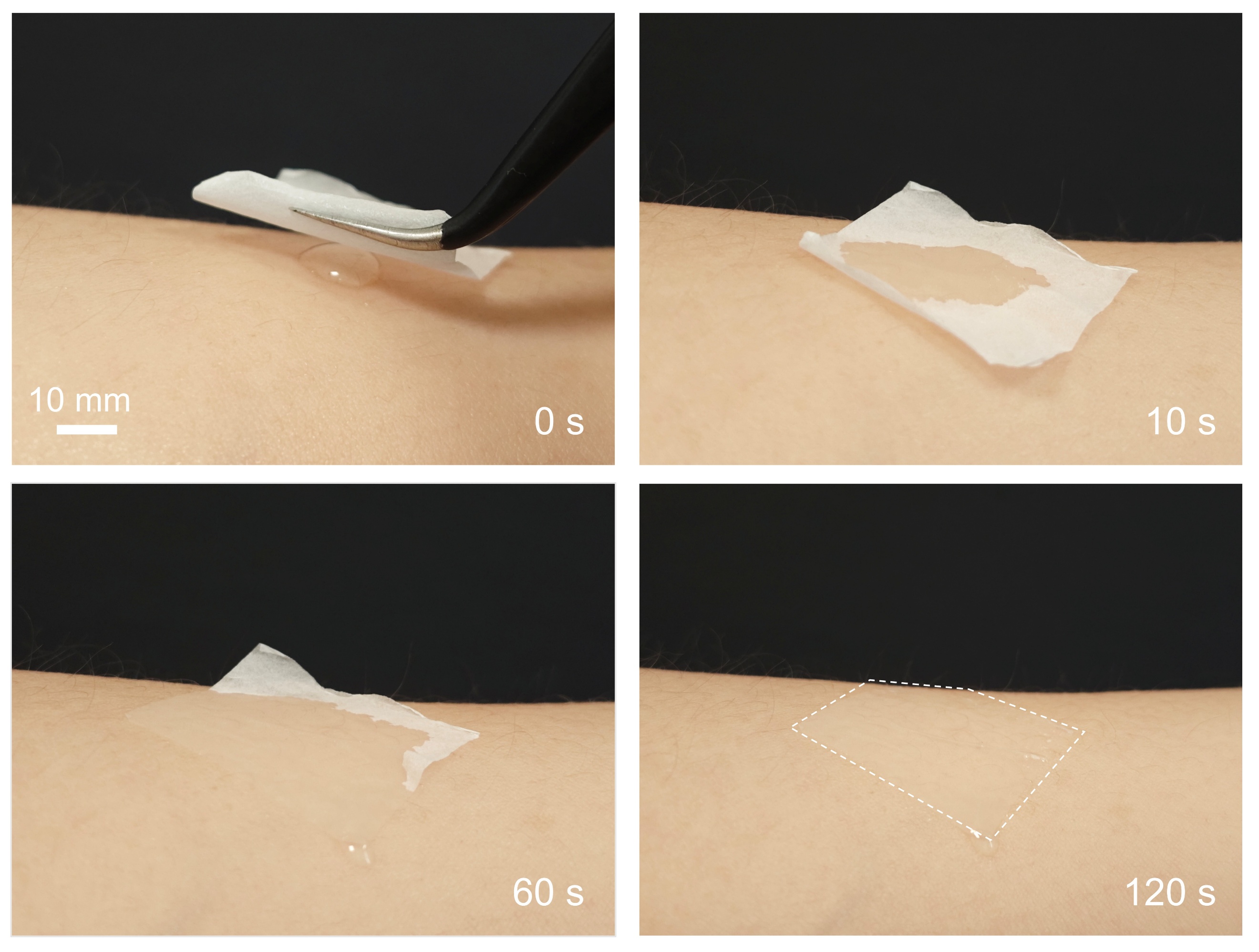
**

**Fig. S11** Photograph of sweat-pumping simulation of SDSN. The figure illustrates the sweat-pumping effect of SDSN simulated by a 50 μL droplet of artificial sweat, which is rapidly drawn to the surface and flows along the edges

**
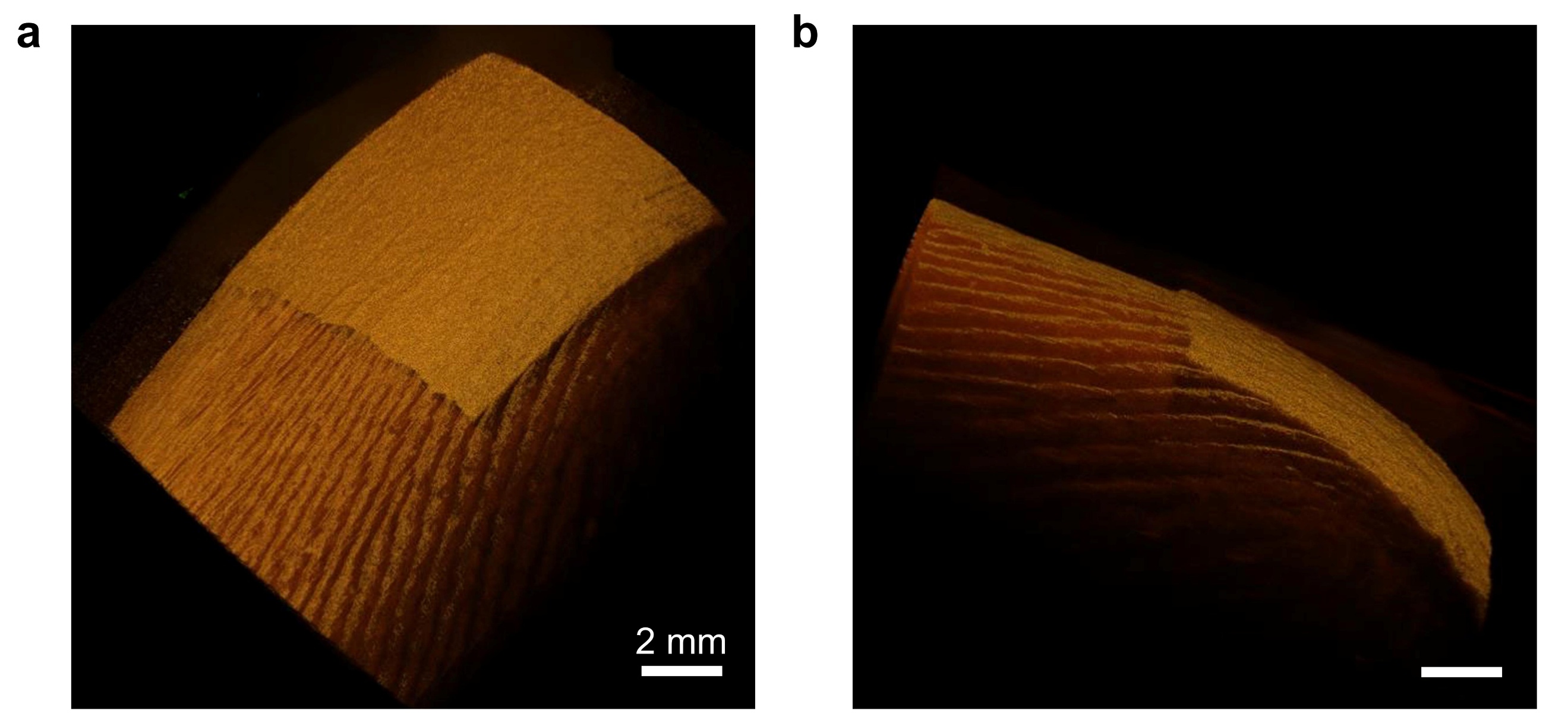
**

**Fig. S12** Optical Coherence Tomography (OCT) images of SDSN attached to the finger. (a) Photo from an oblique upward angle. (b) Photo from a side view

**
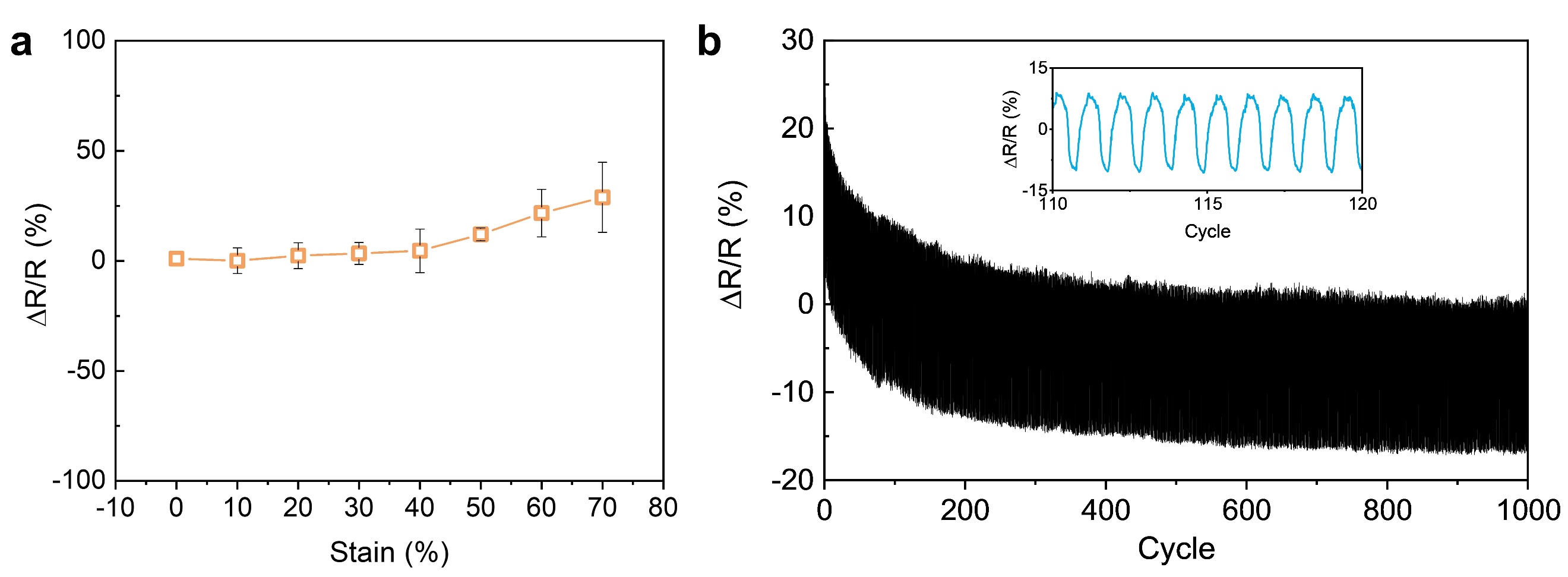
**

**Fig. S13** Stretching stability test of the ANE. (a) Resistance at different stretching lengths. (b) Resistance variation after 1000 stretches at 10% strain


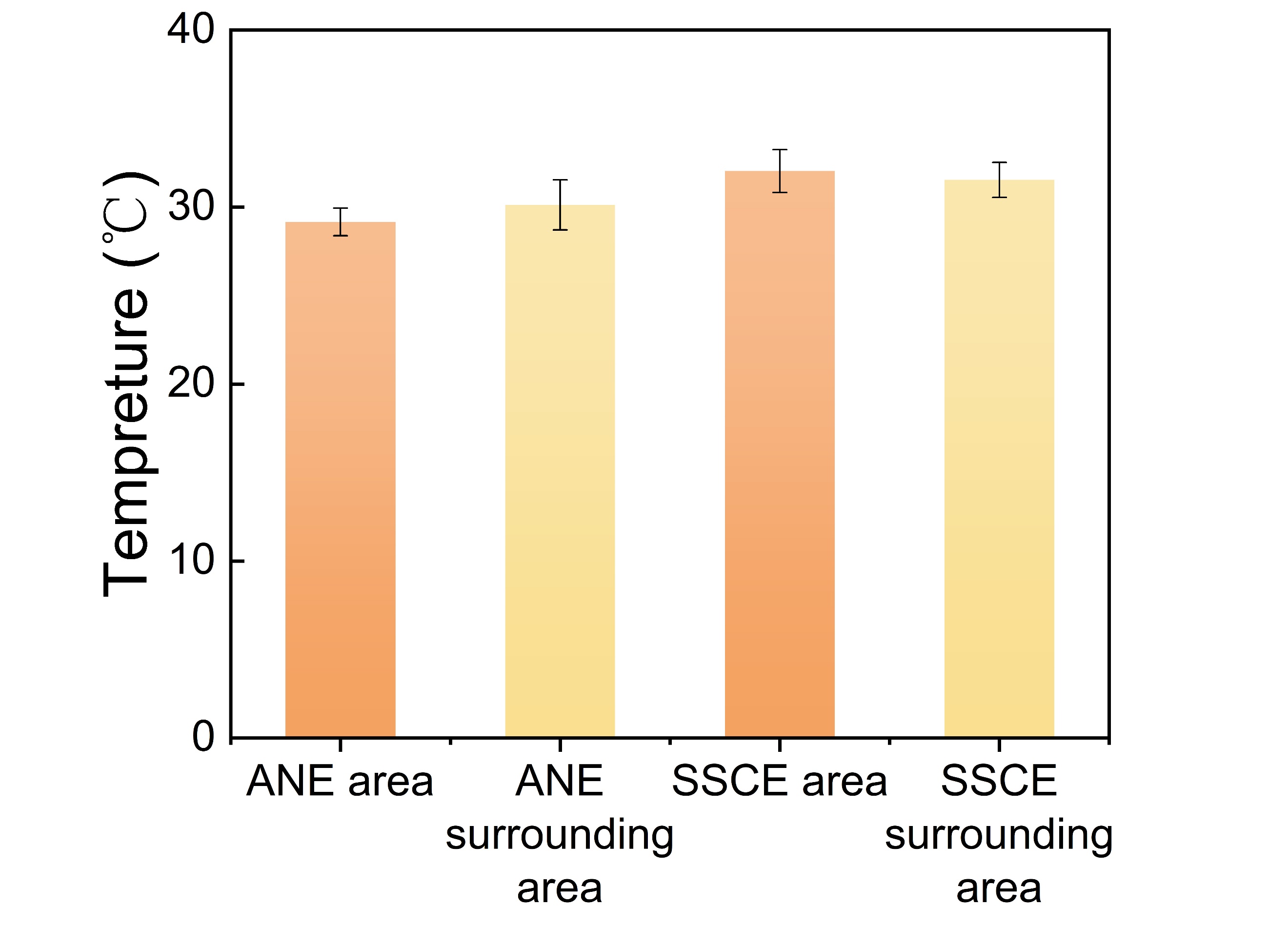


**Fig. S14** Skin temperature under the area covered by the ANE and SSCE and the surrounding bare skin temperature after exercise. Measurements from 10 randomly selected points in the range


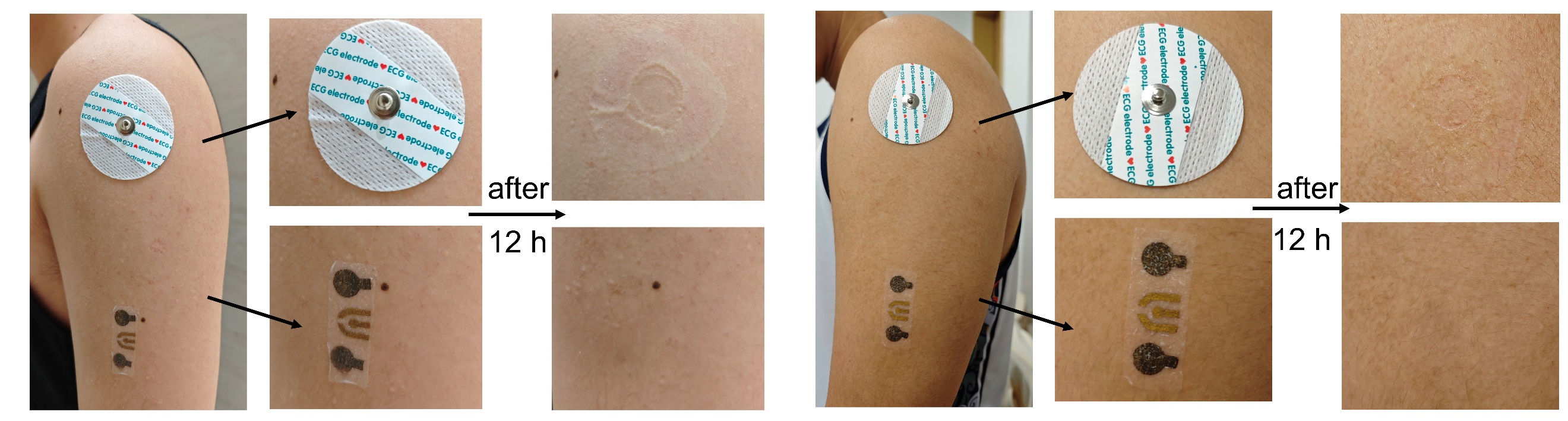


**Fig. S15** The skin irritation test of ANE and SSCE in other subjects. The skin regions covered by SSCE exhibit redness, indicating a mild inflammatory response. In contrast, no color change was observed on the skin covered by ANE, suggesting that it demonstrates excellent biosafety


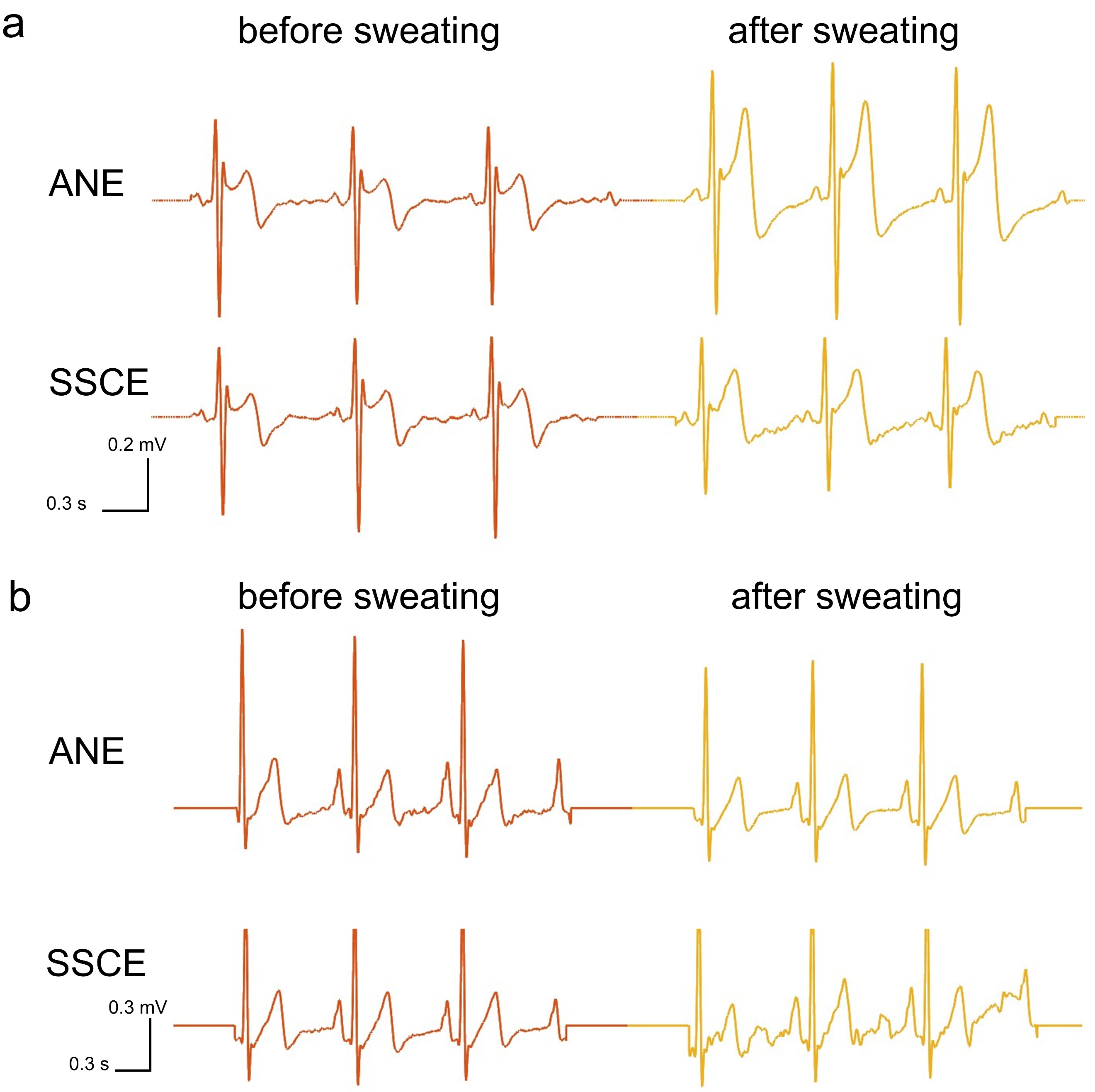


**Fig. S16** ECG measurements before and after sweating for other two participants. (a) The SNRs of the ANE and SSCE are 11.5 dB and 8.9 dB before exercise, and 11.8 dB and 6.4 dB after exercise, respectively. (b) The SNRs of the ANE and SSCE are 9.0 dB and 10.0 dB before exercise, and 10.3 dB and 6.3 dB after exercise, respectively


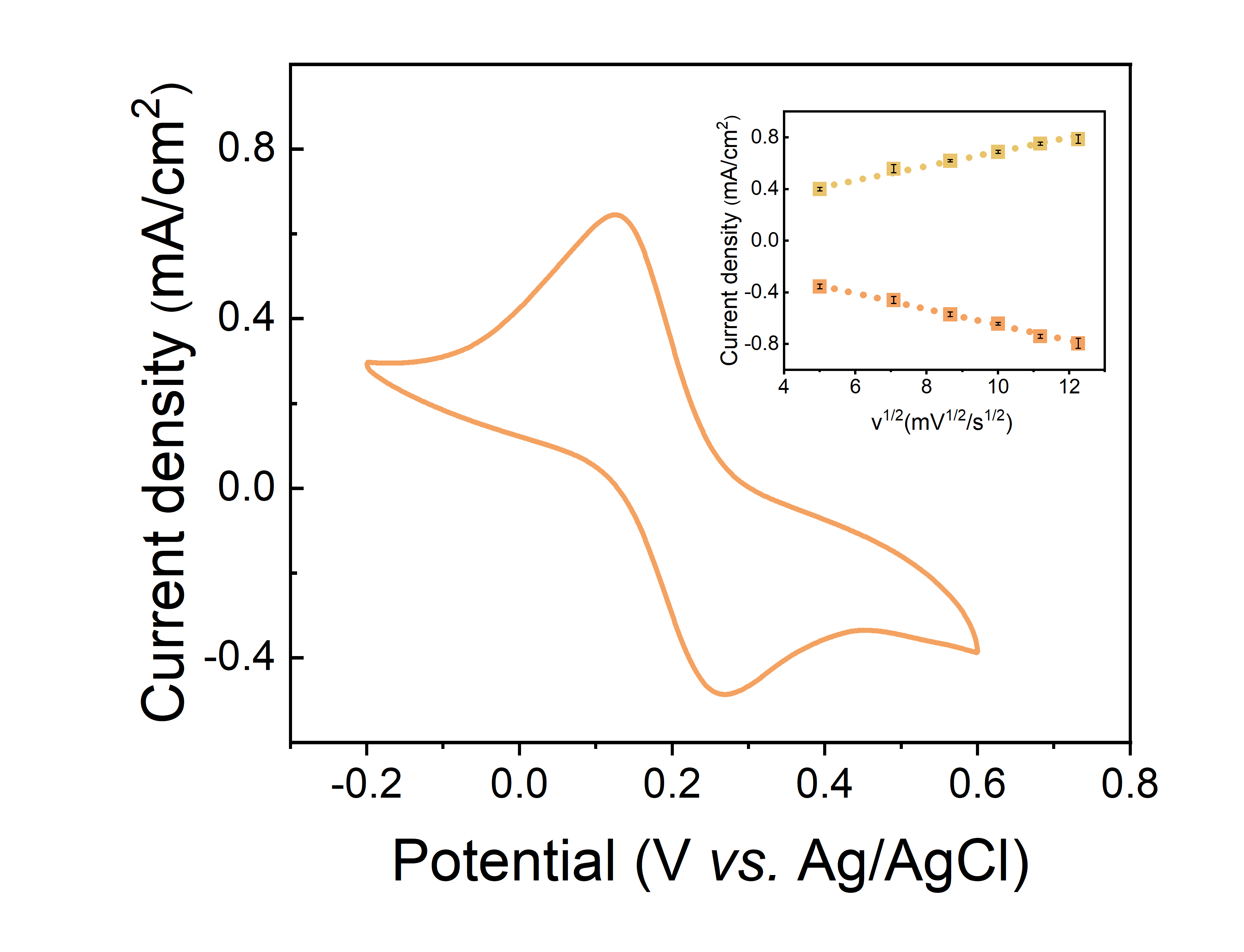


**Fig. S17** Cyclic voltammetry graphs at different scan rates. The solution contains 5 mM K₄[Fe(CN)₆] in PBS. The inset demonstrates the linear regression of the peak current values of anodic and cathodic peaks with the square root of the scan rate

**Fig. S18** Comparison of the slopes of the concentration-current fitting curves at different temperatures. The variations in skin temperature caused by human activities can be considered negligible with respect to their impact on the sensor

**Fig. S19** Current stability of glucose sensors at different pH values. The current with pH =6.5 is regarded as 1.0. The maximum deviation is 2.3% (pH=5.0)

**
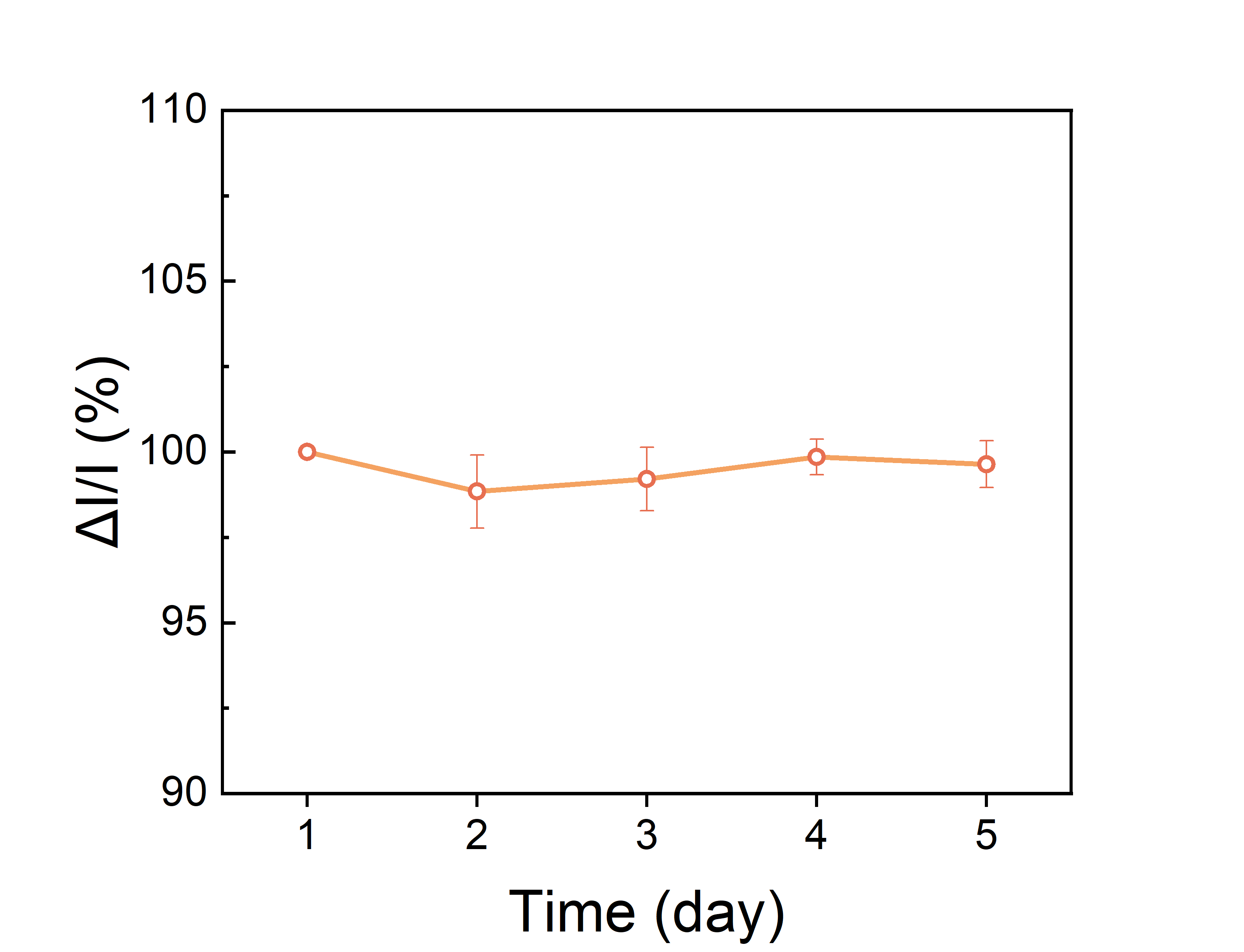
**

**Fig. S20** The change in current for repeated measurements. The analyte is a glucose standard solution at a concentration of 25 μM×L⁻¹. On the fifth day, the change relative to the first day is only 0.36%

**
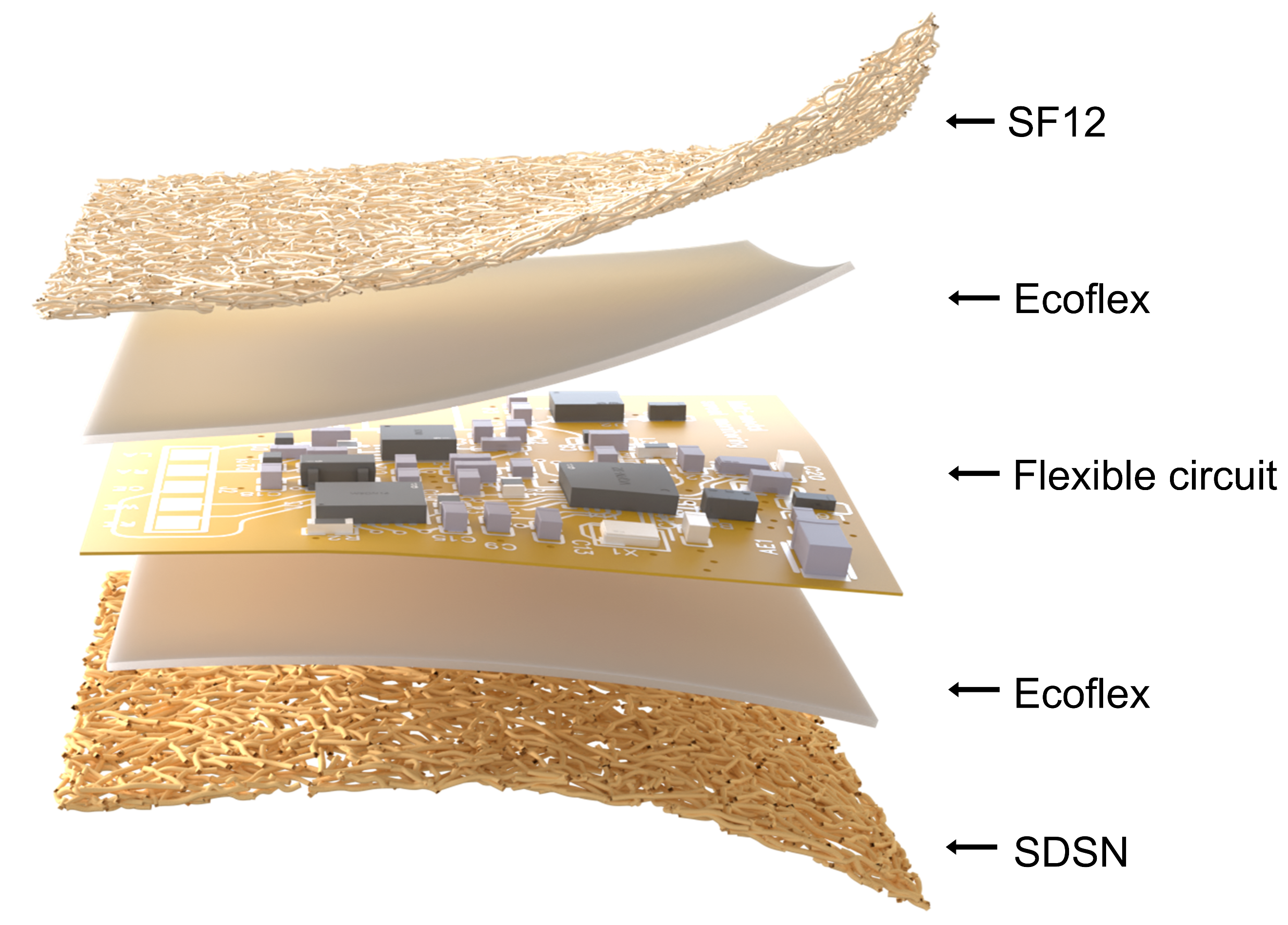
**

**Fig. S21** Schematic diagram of package structure of the circuit. The circuit is encapsulated with Ecoflex to achieve flexible and waterproof packaging. A sandwich structure composed of SDSN and SF12 layers is constructed to impart directional sweat-pumping capability to the entire system


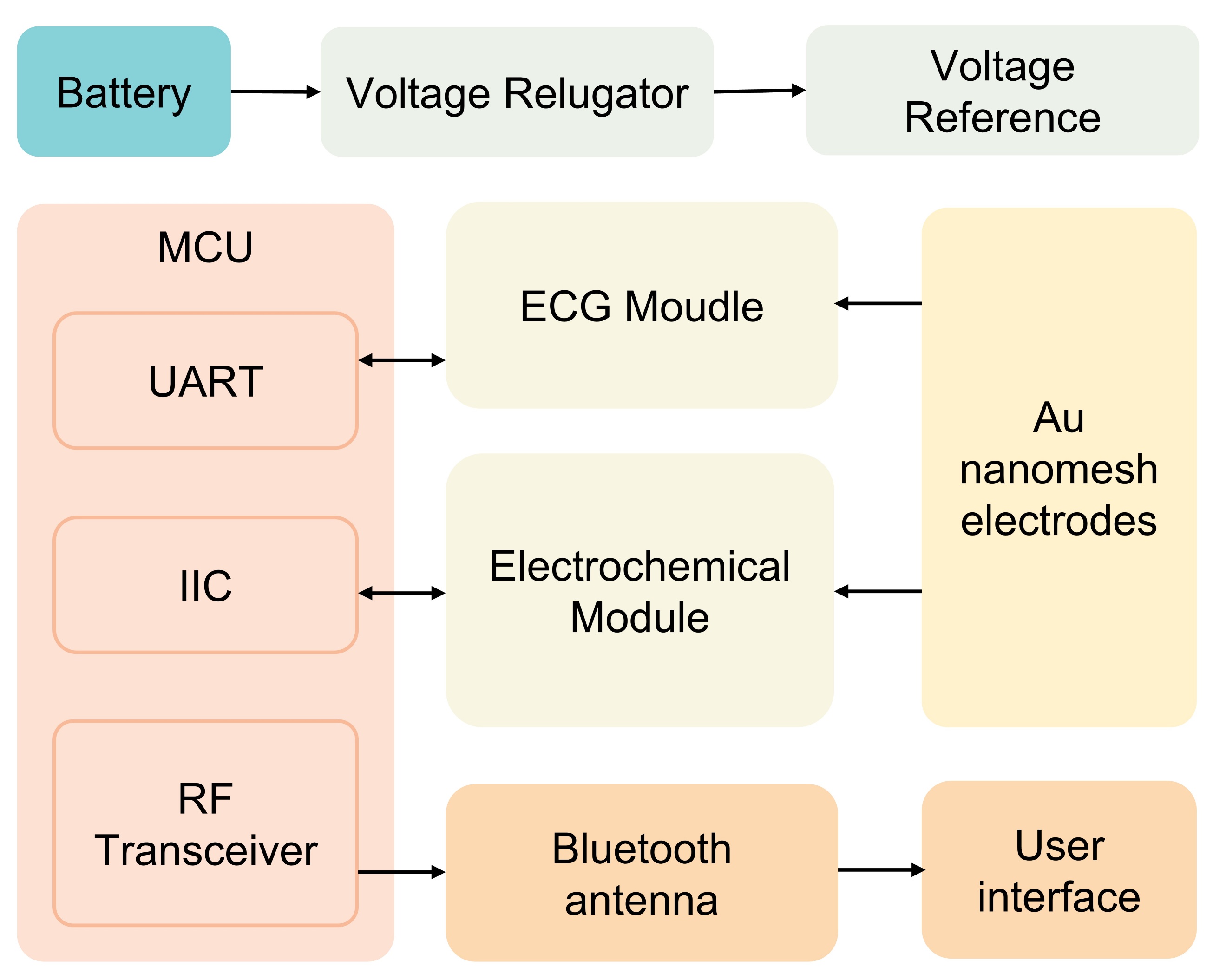


**Fig. S22** Module design of the circuit. Signals are synchronously acquired from the electrochemical and ECG modules and transmitted to the microcontroller unit (MCU), which then wirelessly transmit the data to the host computer via Bluetooth

**
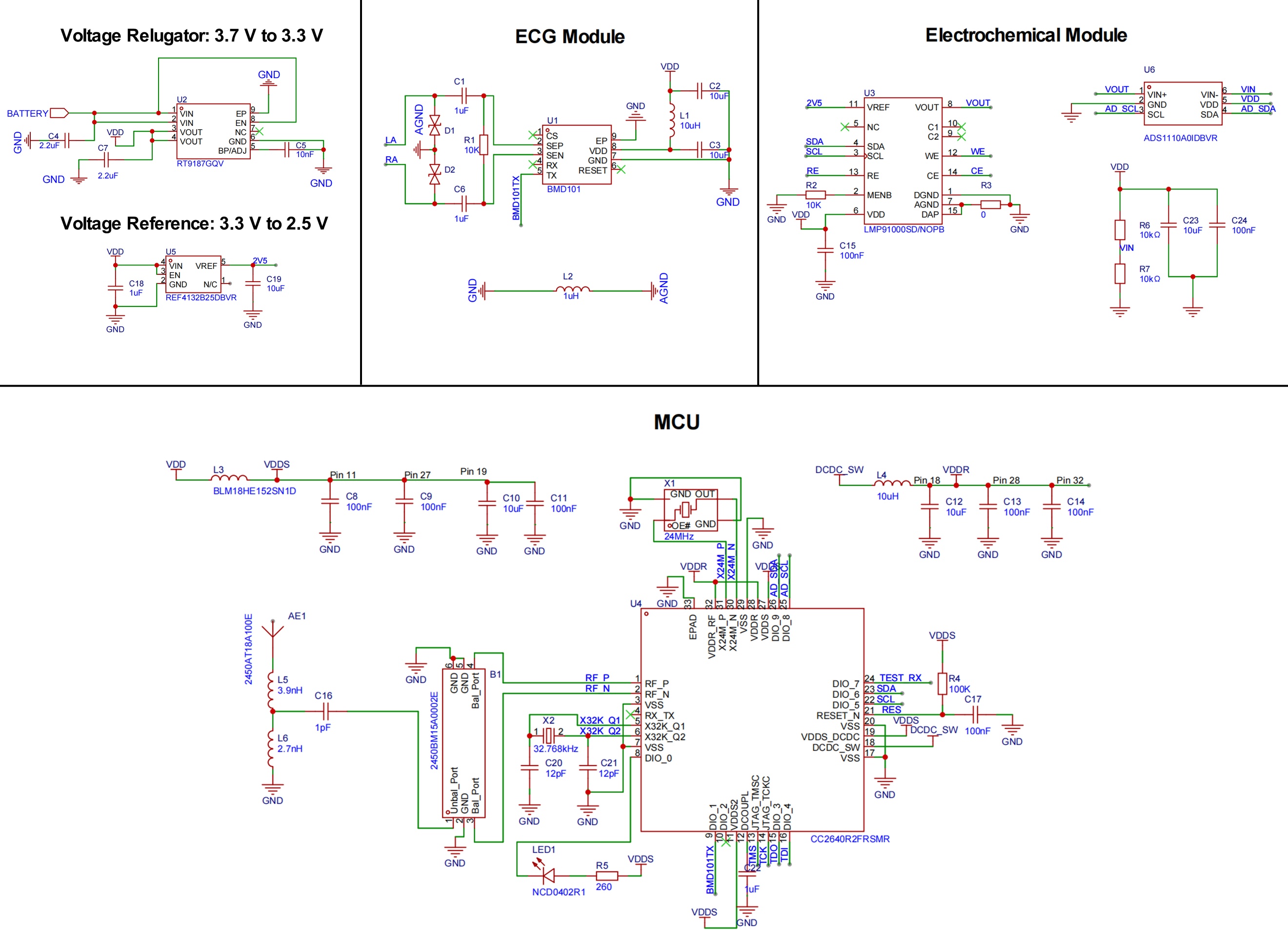
**

**Fig. S23** Schematic diagram of each module of the circuit. The circuit comprises a power management module, a MCU, an ECG module, and an electrochemical module

**
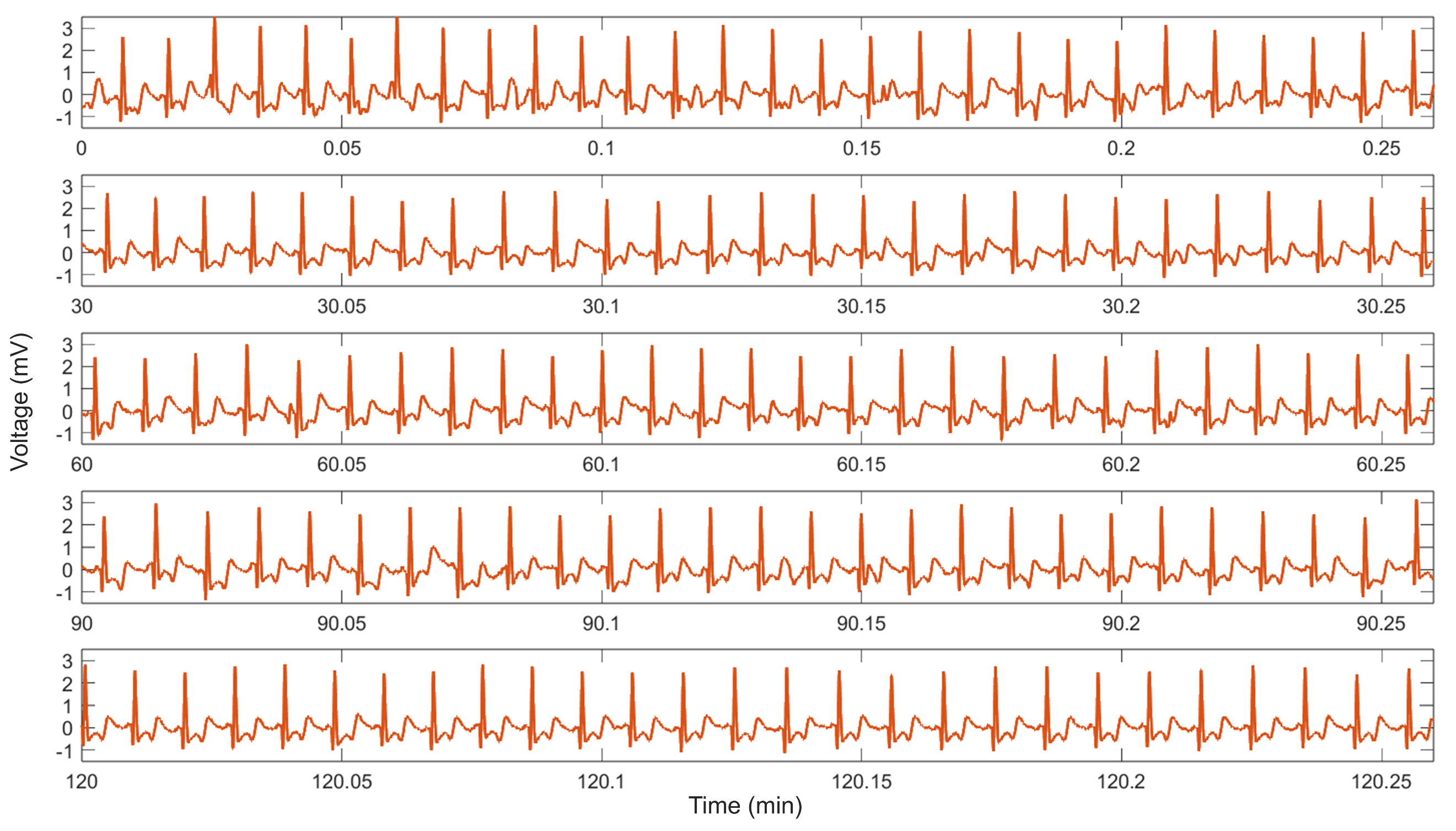
**

**Fig. S24** 2-hour ECG signals continuously monitored by the system


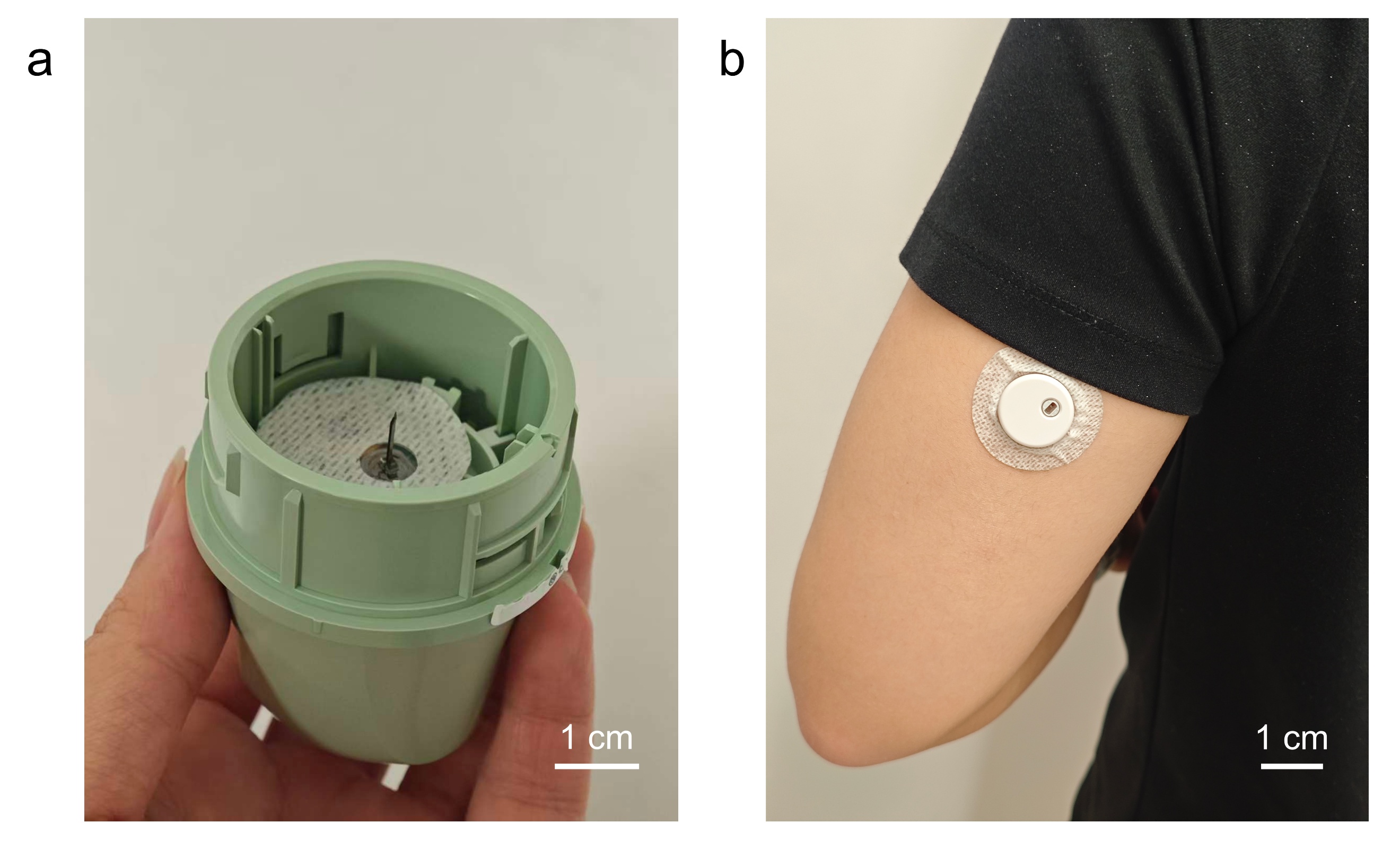


**Fig. S25** Photographs of the continuous glucose monitoring (CGM) device used in test. (a) Internal view of the CGM applicator showing the microneedle, approximately 10 mm in length, intended for subcutaneous insertion. (b) CGM in use on a human subject, with surrounding adhesive tape applied to secure the device


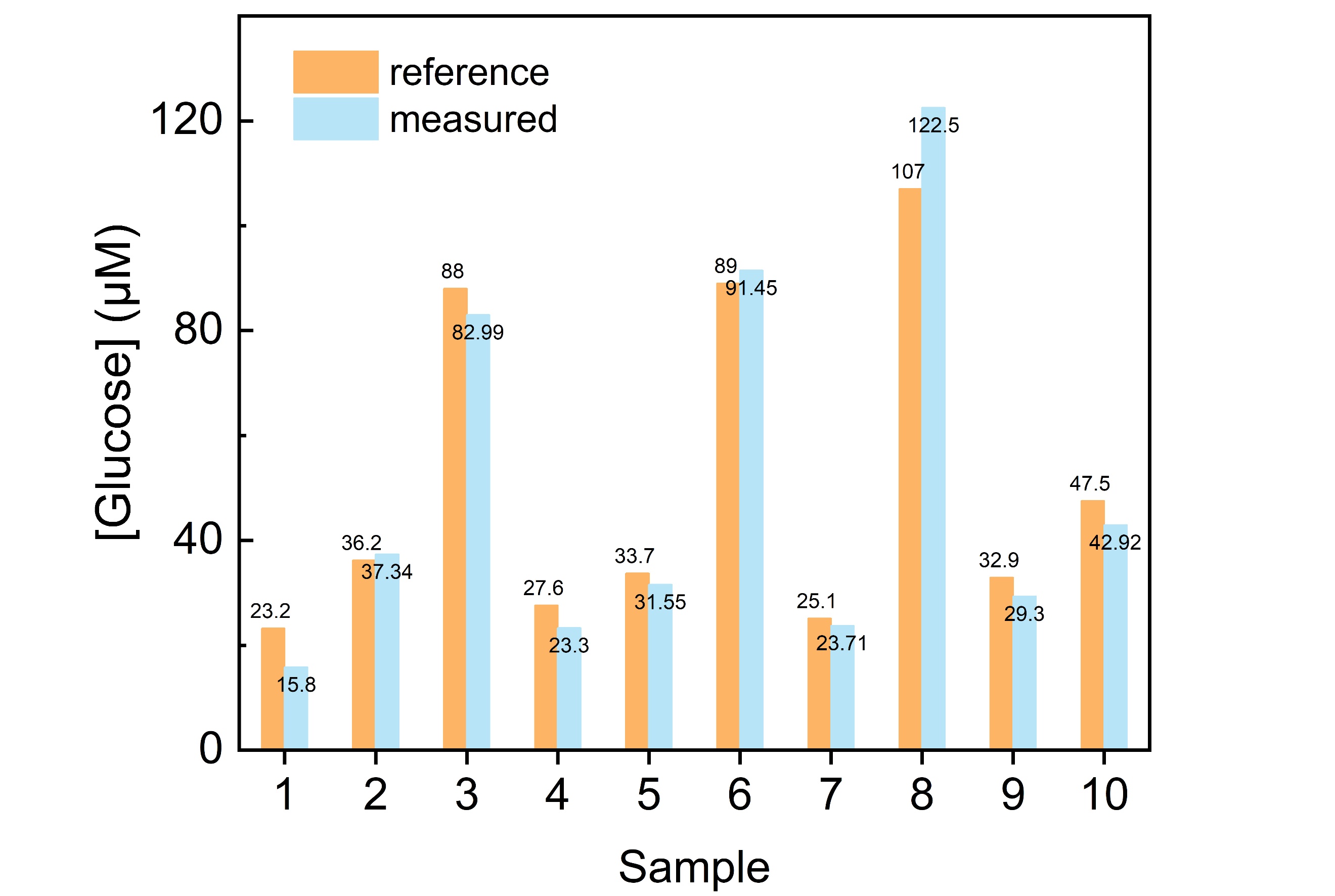


**Fig. S26** Comparison of real/measured glucose level for each sweat sample

**Table S1** Janus characteristics employed in electrospun membranes

| **Year** | **Janus architectures** | | **Liquid uptake rate (μL/s)** | **Sensing function** | | **References** | |
| --- | --- | --- | --- | --- | --- | --- | --- |
| 2018 | | Wettability asymmetry | 5.8 | / | [S3] | |  |
| 2019 | | Wettability asymmetry | 2.7 | / | [S4] | |  |
| 2019 | | Wettability asymmetry | 1.0 | / | [S5] | |  |
| 2019 | | Wettability asymmetry, Pore size asymmetry | 2.0 | / | [S6] | |  |
| 2022 | | Wettability asymmetry, Pore size asymmetry | 0.5 | Electrophysiological | [S7] | |  |
| 2022 | | Wettability asymmetry, Channel diameter asymmetry | 1.6 | Electrophysiological | [S8] | |  |
| 2023 | | Wettability asymmetry | 2.0 | Sweating rate, Sweat ions | [S9] | |  |
| 2023 | | Wettability asymmetry | / | Strain | [S10] | |  |
| 2023 | | Wettability asymmetry | / | Sweat biomarkers | [S11] | |  |
| 2023 | | Wettability asymmetry, Pore size asymmetry, Thermal conductivity asymmetry | 2.0 | / | [S12] | |  |
| 2024 | | Wettability asymmetry, Pore size asymmetry | 1.1 | Strain | [S13] | |  |
| 2025 | | Wettability asymmetry, Pore size asymmetry | 0.5 | Electrophysiological | [S14] | |  |
| 2025 | | Wettability asymmetry, Pore size asymmetry, Thermal conductivity asymmetry | / | Strain | [S15] | |  |
| 2025 | | Wettability asymmetry, Pore size asymmetry, **Fiber structure asymmetry** | **6.25** | **Electrophysiological, Sweat biomarkers** | This work | |  |

**Table S2** Statistical analysis of fiber parameters from five different batches of SF12 nanomesh

|  | Pore size (μm) | Spindle-knots (μm) | | Joints (μm) | Length (μm) |
| --- | --- | --- | --- | --- | --- |
| 1 | 11.83±2.85 | 5.47±1.62 | 1.63±0.57 | | 24.49±4.59 |
| 2 | 15.52±1.75 | 6.86±2.13 | 1.68±0.34 | | 24.77±10.57 |
| 3 | 15.02±2.01 | 5.89±3.22 | 1.57±0.71 | | 25.05±9.27 |
| 4 | 13.64±2.47 | 6.47±1.83 | 1.83±0.49 | | 22.42±5.44 |
| 5 | 12.40±3.83 | 4.32±2.07 | 1.04±0.37 | | 20.83±8.22 |
| Mean | **13.68±2.98** | **5.54±2.53** | **1.43±0.58** | | **23.00±8.28** |

**Table S3** Comparison of this work with recent published permeable electronics**.**

| **Type** | **WVTR** **(g×m^-2^×h^-1^)** | **References** |
| --- | --- | --- |
| SDSN | 132.6 | This work |
| Silk-based electrode | 117 | [S16] |
| PTFE/AgNWs/Silk fabric | 106.4 | [S17] |
| HPP electrode | 102 | [S18] |
| NW-THV-CNTs piezoresistive sensor | 67.7 | [S19] |
| SEBS-based triboelectric sensors | 58.8 | [S20] |
| E-skin patch | 14.5 | [S21] |

**Table S4** Compare the influence of circuit size and the number of hydrophilic layers in the package on the permeability and evaporation of the circuit part

|  | Air permeability (g×m^-2^×h^-1^) | Liquid evaporation (g×m^-2^×h^-1^) |
| --- | --- | --- |
| SDSN | 132.6 | 268 |
| SF0/C1/SF12 | 60.56 | 137.02 |
| SF0/C2/SF12 | 77.3 | 125.31 |
| **SF0/SF12/C2/SF12** | **72.06** | **143.26** |

With total device area fixed, two circuit footprints: C1 = 9 cm^2^ (25.5% coverage) and C2 = 6 cm^2^ (17.0%) are compared and varied the number of hydrophilic encapsulation layers. Higher coverage produced the lowest air permeability (SF0/C1/SF12). Adding a second hydrophilic layer enhanced moisture transport, yielding the highest evaporation rate among circuit-integrated fabrics (SF0/SF12/C2/SF12) with good air permeability.

**Supplementary References**

1. Z. Peng, C. Wang, L. Chen, S. Chen, Peeling behavior of a viscoelastic thin-film on a rigid substrate. Int. J. Solids Struct. **51**(25–26), 4596–4603 (2014). <https://doi.org/10.1016/j.ijsolstr.2014.10.011>
2. T. Nishikawa, H. Yamane, N. Matsuhisa, N. Miki, Stretchable strain sensor with small but sufficient adhesion to skin. Sensors **23**(4), 1774 (2023). <https://doi.org/10.3390/s23041774>
3. D. Miao, Z. Huang, X. Wang, J. Yu, B. Ding, Continuous, spontaneous, and directional water transport in the trilayered fibrous membranes for functional moisture wicking textiles. Small **14**(32), e1801527 (2018). <https://doi.org/10.1002/smll.201801527>
4. L. Shi, X. Liu, W. Wang, L. Jiang, S. Wang, A self-pumping dressing for draining excessive biofluid around wounds. Adv. Mater. **31**(5), e1804187 (2019). <https://doi.org/10.1002/adma.201804187>
5. B. Dai, K. Li, L. Shi, X. Wan, X. Liu et al., Bioinspired Janus textile with conical micropores for human body moisture and thermal management. Adv. Mater. **31**(41), e1904113 (2019). <https://doi.org/10.1002/adma.201904113>
6. X. Wang, Z. Huang, D. Miao, J. Zhao, J. Yu et al., Biomimetic fibrous Murray membranes with ultrafast water transport and evaporation for smart moisture-wicking fabrics. ACS Nano **13**(2), 1060–1070 (2019). <https://doi.org/10.1021/acsnano.8b08242>
7. Y. Xu, W. Guo, S. Zhou, H. Yi, G. Yang et al., Bioinspired perspiration-wicking electronic skins for comfortable and reliable multimodal health monitoring. Adv. Funct. Mater. **32**(23), 2200961 (2022). <https://doi.org/10.1002/adfm.202200961>
8. P. Li, Y. Bao, B. Chen, X. Liu, H. Yang et al., A bioinspired sweat-drainable Janus electrophysiological electrode for scientific sports training. Adv. Mater. Technol. **7**(10), 2200040 (2022). <https://doi.org/10.1002/admt.202200040>
9. M. Liu, S. Wang, Z. Xiong, Z. Zheng, N. Ma et al., Perspiration permeable, textile embeddable microfluidic sweat sensor. Biosens. Bioelectron. **237**, 115504 (2023). <https://doi.org/10.1016/j.bios.2023.115504>
10. C. Zhi, S. Shi, S. Zhang, Y. Si, J. Yang et al., Bioinspired all-fibrous directional moisture-wicking electronic skins for biomechanical energy harvesting and all-range health sensing. Nano-Micro Lett. **15**(1), 60 (2023). <https://doi.org/10.1007/s40820-023-01028-2>
11. P. Xi, X. He, C. Fan, Q. Zhu, Z. Li et al., Smart Janus fabrics for one-way sweat sampling and skin-friendly colorimetric detection. Talanta **259**, 124507 (2023). <https://doi.org/10.1016/j.talanta.2023.124507>
12. C. Fan, Y. Zhang, Z. Long, A. Mensah, Q. Wang et al., Dynamically tunable subambient daytime radiative cooling metafabric with Janus wettability. Adv. Funct. Mater. **33**(29), 2300794 (2023). <https://doi.org/10.1002/adfm.202300794>
13. G. Wang, Z. Xie, W. Yu, S. Mao, S. Wang et al., A double-layer polyurethane electrospun membrane with directional sweat transport ability for use as a soft strain sensor. ACS Appl. Mater. Interfaces **16**(37), 49813–49822 (2024). <https://doi.org/10.1021/acsami.4c10854>
14. J. Li, C. Yang, C. Ma, J. Yang, S. Zhou et al., A self-adhesive breathable Janus fibrous membrane for electronic skins with long-term comfort and reliability. Chem. Eng. J. **522**, 166859 (2025). <https://doi.org/10.1016/j.cej.2025.166859>
15. Y. Chen, Y. Jiang, X. Zhang, T. Zhang, W. Feng et al., Janus nanofibrous membranes for multifunctional sensing and personal thermal-moisture management. Chem. Eng. J. **519**, 165413 (2025). <https://doi.org/10.1016/j.cej.2025.165413>
16. Q. Li, G. Chen, Y. Cui, S. Ji, Z. Liu et al., Highly thermal-wet comfortable and conformal silk-based electrodes for on-skin sensors with sweat tolerance. ACS Nano **15**(6), 9955–9966 (2021). <https://doi.org/10.1021/acsnano.1c01431>
17. X. Yan, S. Chen, G. Zhang, W. Shi, Z. Peng et al., Highly breathable, surface-hydrophobic and wet-adhesive silk based epidermal electrode for long-term electrophysiological monitoring. Compos. Sci. Technol. **230**, 109751 (2022). <https://doi.org/10.1016/j.compscitech.2022.109751>
18. C. Ma, S. Hao, W. Yu, X. Liu, Y. Wang et al., Compliant and breathable electrospun epidermal electrode towards artifact-free electrophysiological monitoring. Chem. Eng. J. **490**, 151118 (2024). <https://doi.org/10.1016/j.cej.2024.151118>
19. L. Li, X. Zhou, B. Jin, K. Hou, D. Yu et al., Highly compressible, breathable, and waterproof piezoresistive sensors based on commercial three-dimensional air-laid nonwovens. Colloid Polym. Sci. **302**(3), 449–461 (2024). <https://doi.org/10.1007/s00396-023-05207-w>
20. K. Shrestha, G.B. Pradhan, M.S. Raza, J.Y. Park, A porous SEBS-based breathable triboelectric nanogenerator for human gesture recognition. 2024 IEEE 37th International Conference on Micro Electro Mechanical Systems (MEMS)., 105–108. IEEE (2024).
21. W. Hwang, J. Kim, S. Park, T.-H. Kang, S. Kim et al., A breathable and stretchable metastructure for a versatile hybrid electronic skin patch with long-term skin comfort. Adv. Mater. Technol. **8**(1), 2200477 (2023). <https://doi.org/10.1002/admt.202200477>
